# A non-GMO *Saccharomyces cerevisiae* × *Saccharomyces paradoxus* brewing hybrid moderately reduces ethanol production and modifies beer aroma

**DOI:** 10.3389/fmicb.2026.1948430

**Published:** 2026-09-29

**Authors:** David Roldán-López, Isabel E. Sánchez-Adriá, Elizabeth Carrillo, Sandra Sánchez-Martí, Roberto Pérez-Torrado

**Affiliations:** Instituto de Agroquímica y Tecnología de Alimentos (IATA-CSIC), València, Spain

**Keywords:** aroma, brewing, interspecific hybrid, non-GMO, reduced alcohol beer, yeast

## Abstract

The increasing demand for beers with moderate ethanol content and diversified sensory profiles has stimulated the search for non-GMO yeast-based strategies that preserve brewing performance. In this study, a collection of 28 yeast strains from different species and isolation sources was screened to identify parental strains with complementary fermentative and metabolic traits. Based on their performance under brewing conditions, selected parents were crossed by rare-mating to generate a novel interspecific hybrid, DR1 (*Saccharomyces cerevisiae* × *Saccharomyces paradoxus*), which was molecularly confirmed and subsequently characterized. The fermentative behavior of DR1 was assessed in comparison with its parental strains under representative and industrial brewing conditions. DR1 retained efficient maltose consumption but showed limited maltotriose utilization, resulting in a moderate ethanol reduction relative to the industrial parent, reaching 21 % in synthetic wort and 17.5 % in industrial wort. The hybrid also produced higher glycerol and a volatile profile enriched in selected acetate and fatty acid esters. Consumer sensory analysis showed that DR1 retained beer-like sensory attributes, although overall liking and purchase intention were lower than for the industrial parent, indicating that further optimization will be required for broad consumer acceptance. Overall, this work supports rare-mating-based interspecific hybridization as a non-GMO strategy to generate brewing yeasts with reduced ethanol production and expanded aromatic diversity.

## 1 Introduction

The beer industry is undergoing a transformation driven by a change in consumer preferences toward products with innovative sensory profiles and reduced alcohol content (Krogerus et al., 2017). Yeast is the main player in beer production for two main reasons.

First, primary metabolism: brewer’s wort contains a high concentration of carbohydrates, including fermentable sugars and non-fermentable dextrins, in relatively consistent proportions. Maltose is the main fermentable sugar, typically accounting for 30–70% of the total wort carbohydrates, whereas maltotriose and glucose generally represent 0.5–25% each. Fructose and sucrose are present in lower amounts (1–10%), while the remaining carbohydrate fraction consists mainly of non-fermentable dextrins (polymeric α-glucosides), which cannot be metabolized by conventional brewing *Saccharomyces* strains and therefore contribute to beer body rather than ethanol production (Aredes et al., 2025; Wasmuht, 2023).

Wort sugar composition strongly influences ethanol production by brewing yeasts. Glucose, which is present at relatively low concentrations, is readily taken up by Hxt hexose transporters (Donzella et al., 2023; Kim et al., 2013). In contrast, maltose and maltotriose, the main fermentable sugars in wort, require active uptake through α-glucoside proton symporters encoded by genes such as MALx1, AGT1, MTT1 and MPHX, followed by intracellular hydrolysis into glucose by α-glucosidases (Novak et al., 2004; Vidgren and Londesborough, 2018). Therefore, differences in maltose and especially maltotriose utilization largely depend on the presence, copy number and substrate specificity of MAL loci and associated transporters (Horák, 2013; Vidgren and Londesborough, 2018). This variability is particularly evident between group I (Saaz) and group II (Frohberg) lager strains, with the former generally showing limited maltotriose utilization and the latter efficiently fermenting this sugar (Donzella et al., 2023).

Second, secondary metabolism: Yeast secondary metabolism also plays a central role in beer aroma, mainly through the production of volatile organic metabolites such as higher alcohols, acetate esters, fatty acid esters, aldehydes, acids and volatile phenols (Dzialo et al., 2017). Higher alcohols and esters contribute fruity and floral notes, whereas some compounds, including vicinal diketones, sulfur compounds and volatile phenols, can be undesirable depending on concentration and beer style (Holt et al., 2019; Krogerus and Gibson, 2013; van Bergen et al., 2016). In particular, 4-vinylguaiacol and 4-vinylphenol are generally considered phenolic off-flavors in lager beers but can contribute positively to wheat and Belgian-style beers (Vanbeneden et al., 2008). Therefore, yeast strain selection and hybridisation can modulate both ethanol production and aroma complexity.

For decades, beer production has been based on an extremely limited number of domesticated strains of *Saccharomyces cerevisiae* and *Saccharomyces pastorianus*, resulting in genetic homogeneity that restricts the phenotypic innovation needed to meet current healthy consumption trends (Gallone et al., 2018; Lodolo et al., 2008). In this context, the use of non-conventional yeasts and the generation of interspecific hybrids are presented as key biotechnological strategies for product diversification. Interspecific hybrids have been isolated from different fermented beverages, including wine, cider and beer (Krogerus et al., 2017). The most notable example is *S. pastorianus*, which is a hybrid of *S. cerevisiae* and *S. eubayanus*, containing both subgenomes (Cousseau et al., 2013).

In most cases, hybrids acquire interesting combinations of physiological characteristics from parental strains, being tools for specific technological uses. For this reason, many artificial hybrid yeasts have been constructed in recent decades to improve different metabolic behaviors related to beer production. This technique has been used to improve the fermentation rate and ester formation (Mukai et al., 2001), to improve sugar utilization and fermentation rate (Hebly et al., 2015), to increase the production of aroma compounds (Krogerus et al., 2015), or to improve the aroma complexity (Mertens et al., 2015). They are also used in wine production, hybrids created between *S. cerevisiae, S. kudriavzevii, S. mikatae, S. paradoxus* and *S. uvarum* have shown the potential to create a wide aromatic diversity in the wine industry (Lopandic et al., 2016).

Those examples demonstrate that interspecific hybridisation is one of the best tools for non-GMO yeast strain development, allowing the combination and enhancement of phenotypic traits from different parental strains (Bellon et al., 2015).

The development of hybrids is executed via three primary methodologies: spore–spore mating, rare mating, and protoplast fusion. Spore-to-spore mating is based on the crossing of haploid spores of the opposite sex. It has a high success rate, does not require selection markers, but requires both parents to sporulate well. Protoplast fusion is based on the deletion of the cell wall from both parental cells and the fusion of the protoplasts before the cell wall is regenerated. It allows sexually incompatible genera or species to be combined but is highly genomically unstable. Finally, rare-mating, the one used in this work, is based on the spontaneous loss of heterozygosity at the MAT locus. It is a useful option for overcoming problems with low sporulation, but it is an uncommon event, which requires the use of selection markers and produces hybrids with a high chromosome number (Winans, 2022).

However, depending on the hybridization strategy employed, newly generated interspecific hybrids may require several generations to achieve genetic stability. In particular, hybrids obtained by rare mating often display different degrees of genomic instability and may require stabilization before industrial application (Pérez-Través et al., 2014). In order to use this technique, it is necessary to find yeasts with interesting traits that we want to hybridize with another yeast.

For the generation of interspecific hybrids for use in the brewing industry, a strain of *S. cerevisiae* is often selected as the parent, as it is one of the two parents of *S. pastorianus*. The choice of the parental lineage of *S. cerevisiae* is critical in defining both the aroma and the ability to process the sugars in beer (Zavaleta et al., 2024). The other parent is selected with the aim of incorporating a distinctive characteristic. In *S. pastorianus*, the second parental strain is a *S. eubayanus* strain, which provides cold tolerance.

The hybridisation of *S. cerevisiae* with *S. arboricola*, *S. mikatae* and *S. uvarum* has shown that similar results can be achieved with the use of species other than *S. eubayanus*, providing increased diversity with novel organoleptic profiles (Nikulin et al., 2018). Wild yeasts of *S. paradoxus* have recently been shown to have potential for use in the brewing industry, due to their fermentation performance and the distinctive aromas they impart when fermenting at low temperatures (Nikulin et al., 2020).

In this study, an initial screening of 28 yeast strains of various species and origins was carried out to evaluate their fermentation kinetics and metabolic profile in synthetic beer wort (supplemented with ZnSO_4_ and iso-α-acids in order to emulate brewing conditions). Based on these results, parents with complementary traits were selected to generate new hybrids through rare-mating. The aim of this project was to generate novel yeast hybrids with reduced ethanol production and an enhanced aroma profile, while preserving fermentative performance, through rare-mating techniques, and to evaluate the resulting beers using analytical and sensory analyzes.

## 2 Materials and methods

### 2.1 Parental yeast strains collection

A collection of 28 yeast strains was used as potential parents for hybrid generation (Table 1). This collection includes 20 strains belonging to the genus *Saccharomyces*, divided into: 14 strains of *S. cerevisiae*, 2 strains of *S. paradoxus*, 2 strains of *S. mikatae* and 2 strains of *S. arboricola*. In addition, 6 hybrids of the genus *Saccharomyces* have been included: 2 hybrids of *S. cerevisiae* x *S. eubayanus* (*S. pastorianus*), 2 hybrids of *S. cerevisiae* x *S. kudriavzevii* and 2 hybrids of *S. eubayanus* x *S. uvarum*. Finally, 2 strains belong to the genus *Kazachstania*: 1 strain of *K. servazii* and 1 strain of *K. bovina*. The commercial strain of *S. pastorianus* Saflager Weihenstephan 34/70 (WS 34/70) was used as a commercial control during the study. The *S. cerevisiae* strains include 3 industrial strains (Lal 1, Lal 2 and Lal 3) related to beer production. Strains were isolated from a variety of sources, including fermentative environments such as beer, wine, or bioethanol, as well as non- fermentative environments like animals, fruits, or trees. The yeasts were cryopreserved at −80 °C. For their use, the strains were grown and maintained on solid GPY medium plates (2% glucose, 2% agar, 0.5% peptone, 0.5% yeast extract) and stored at 4 °C.

**TABLE 1:** Strain information and kinetic parameters of fermentation for the 28 wild-type yeast strains screened as potential parents for hybrid generation under brewing conditions.

Code	Strain	Isolation source	Species	Subpopulation	AUC	A (g/L)	μ max (h_–1_)	λ (h)	References
WS 34/70	WS 34/70	Beer	*S. cerevisiae x S. eubayanus (S. pastorianus)*		6.47 ± 0.49 x 10^3^	4.65 ± 0.36 x 10^1^	1.26 ± 0.07	2.14 ± 0.22 x 10^1^	Roldán-López et al., 2025
Lal 1	Lal 1	Beer	*S. cerevisiae*		6.82 ± 0.43 x 10^3^	4.69 ± 0.22 x 10^1^	1.29 ± 0.05	2.26 ± 0.25 x 10^1^	This study
Lal 2	Lal 2	Beer	*S. cerevisiae*		6.09 ± 0.19 x 10^3^	4.77 ± 0.04 x 10^1^	6.63 ± 0.86 x 10^−1^	1.92 ± 0.25 x 10^1^	
Lal 3	Lal 3	Beer	*S. cerevisiae*		6.51 ± 0.29 x 10^3^	4.28 ± 0.20 x 10^1^	1.95 ± 0.04	2.29 ± 0.05 x 10^1^	
BID	DBVPG1058	Bakery	*S. cerevisiae*	Wine/European	5.86 ± 0.30 x 10^3^	3.89 ± 0.21 x 10^1^	1.82 ± 0.07	2.15 ± 0.05 x 10^1^	Peter et al., 2018
ATR	CECT10120	Fruit	*S. cerevisiae*	Mixed origin	6.68 ± 0.04 x 10^3^	4.68 ± 0.03 x 10^1^	1.33 ± 0.08	1.94 ± 0.05 x 10^1^	
CDI	N163.01-5A	Tree	*S. cerevisiae*	Far East Russian	6.31 ± 0.41 x 10^3^	6.19 ± 1.82 x 10^1^	1.30 ± 0.01 x 10^−1^	3.02 ± 1.56 x 10^1^	
BVA	RP.10.4	Bioethanol	*S. cerevisiae*	Brazilian bioethanol	6.18 ± 0.04 x 10^3^	4.19 ± 0.02 x 10^1^	1.38 ± 0.08	1.81 ± 0.09 x 10^1^	
BBG	CCY_21-4-106	Water	*S. cerevisiae*	Ale beer	6.17 ± 0.19 x 10^3^	4.60 ± 0.15 x 10^1^	1.01 ± 0.02	1.99 ± 0.06 x 10^1^	
CGD	UCD_09-448	Fruit	*S. cerevisiae*	Alpechin	5.66 ± 0.08 x 10^3^	3.96 ± 0.08 x 10^1^	1.19 ± 0.07	2.13 ± 0.08 x 10^1^	
CIN	malade Pac6436	Human, clinical	*S. cerevisiae*	Wine/European (subclade 3)	5.45 ± 0.26 x 10^3^	3.84 ± 0.17 x 10^1^	1.05 ± 0.07	1.95 ± 0.08 x 10^1^	
CPG	1560	Nature	*S. cerevisiae*	Alpechin	5.11 ± 0.34 x 10^3^	3.63 ± 0.27 x 10^1^	4.08 ± 0.21 x 10^−1^	1.80 ± 0.42 x 10^1^	
CLB	sample 40	Tree	*S. cerevisiae*	Mediterranean oak	3.03 ± 0.86 x 10^3^	2.86 ± 0.80 x 10^1^	1.65 ± 0.56	3.11 ± 0.15	
BAA	DJ66	Palm wine	*S. cerevisiae*	African palm wine	2.73 ± 0.21 x 10^3^	2.66 ± 0.33 x 10^1^	1.41 ± 0.05	4.01 ± 0.47	
CEG	EM14S01-3B	Soil	*S. cerevisiae*	Taiwanese	1.24 ± 0.18 x 10^3^	9.12 ± 1.57	2.77 ± 0.58 x 10^−1^	3.49 ± 1.14	
AQ 3504	LL2012-018 = yHDPN160	Tree	*S. paradoxus*	Mix (Am C x Am B)	2.53 ± 0.48 x 10^3^	3.07 ± 0.57 x 10^1^	2.09 ± 0.43	3.56 ± 0.13	Leducq et al., 2016
AQ 3503	LL2012-016	Tree	*S. paradoxus*	America C	9.93 ± 0.19 x 10^2^	7.15 ± 0.22	2.70 ± 0.57 x 10^−1^	5.13 ± 1.77	
AQ 3272	yHAB415	Bark	*S. mikatae*	Asia B	1.35 ± 0.22 x 104	5.51 ± 0.31 x 10^1^	3.96 ± 1.77 x 10^−1^	-	Roldán-López et al., 2025
AQ 3276	yHAB420	Bark	*S. mikatae*	Asia B	3.56 ± 0.10 x 10^3^	3.41 ± 0.07 x 10^1^	1.63 ± 0.13	2.81 ± 0.08	
AQ 1905	CBS 10644	Tree	*S. arboricola*	Asia A	2.50 ± 0.46 x 10^3^	3.04 ± 0.54 x 10^1^	2.12 ± 0.61	3.93 ± 0.57	
AQ 3196	yHAB338	Bark	*S. arboricola*	Asia A	1.31 ± 0.06 x 10^3^	9.37 ± 0.37	3.48 ± 0.32 x 10^−1^	4.27 ± 1.79	
AQ 104	CECT 1463	Beer	*S. cerevisiae x S. eubayanus (S. pastorianus)*		6.80 ± 0.31 x 10^3^	4.93 ± 0.22 x 10^1^	1.18 ± 0.06	2.65 ± 0.09 x 10^1^	
AQ 1170	CECT 11002	Beer	*S. cerevisiae x S. kudriavzevii*		7.97 ± 0.17 x 10^3^	4.72 ± 0.11 x 10^1^	1.43 ± 0.05	2.12 ± 0.09 x 10^1^	
AQ 1078	CECT 11002b	Beer	*S. cerevisiae x S. kudriavzevii*		6.89 ± 0.03 x 10^3^	4.80 ± 0.01 x 10^1^	1.37 ± 0.04	2.46 ± 0.03 x 10^1^	
AQ 110	NBRC 1948 (CECT 11185)	Spoiled beer	*S. eubayanus x S. uvarum*		6.80 ± 0.46 x 10^3^	4.62 ± 0.30 x 10^1^	1.19 ± 0.10	1.93 ± 0.04 x 10^1^	
AQ 1196	CBS 424	Fruit	*S. eubayanus x S. uvarum*		5.55 ± 0.24 x 10^3^	3.89 ± 0.19 x 10^1^	8.66 ± 0.41 x 10^−1^	1.70 ± 0.07 x 10^1^	
AQ 3911	P1J10	Wheat	*K. servazii*		7.03 ± 0.16 x 10^3^	4.75 ± 0.10 x 10^1^	1.54 ± 0.05	1.93 ± 0.11 x 10^1^	
AQ 3936	CBS 6264	Animal	*K. bovina*		7.81 ± 1.00 x 10^2^	7.58 ± 0.99	2.02 ± 0.43 x 10^−1^	1.19 ± 0.27 x 10^1^	

Fermentation kinetics were determined by fitting CO_2_ production data to the reparametrized Gompertz equation. Values represent the mean ± standard deviation of three independent biological replicates (*n* = 3). AUC, area under the fermentation curve; A, maximum CO_2_ production (g/L); μmax, maximum CO_2_ production rate (h_–1_); and λ, lag time (h). The “Reference” column indicates the first publication in which each strain was described or reported. All kinetic parameters presented in this table were experimentally determined in the present study. The strains included in the initial screening were wild-type strains with no known auxotrophies or targeted gene deletions.

### 2.2 Beer wort preparation

A synthetic brewing wort was prepared according to the protocol described in (Holt et al., 2018). For this purpose, Brewferm’s 8 EBC blonde malt extract was used at a concentration of 200 g/L supplemented with ZnSO_4_ at a final concentration of 0.5 mg/L. Each component was weighed on the VWR 611-2236 balance (VWR International, Radnor, PA, USA). The pH was adjusted to 4.5 to reproduce the acidic conditions typically encountered during brewing fermentation and to standardize the fermentation environment for all strains. After autoclaving (110 °C for 15 min), the wort was allowed to settle overnight, and the sediment was discarded. Hops were added by filtration as isomerized hop extract (Brewferm; 94% water and 6% potassium salts of iso-α-acids) at a final concentration of 25 mg/L. In addition to determining beer bitterness, iso-α-acids play an important technological role by enhancing foam stability and by acting as natural preservatives against Gram+ bacteria, contributing to both the physical stability and microbiological safety of beer (De Clippeleer et al., 2014; Schurr et al., 2015). Finally, the sugar concentration in the wort was adjusted to 15.5 ± 0.1 °Bx and the resulting wort was stored at −20 °C in 1 L bottles prepared for use in the fermentation experiments.

### 2.3 Fermentation experiments

Fermentations in wort medium (15.5 ± 0.1 °Bx, pH 4.5, 0.5 mg/L ZnSO_4_, 25 mg/L hops) with the 28 yeast strains were performed in triplicate. Starter cultures were prepared in flasks with 15 mL wort (15.5 ± 0.1 °Bx, pH 4.5, 0.5 mg/L ZnSO_4_, 25 mg/L hops) at 28 °C for 24 h. 100 mL bottles containing 70 mL of wort were inoculated with starter cultures to a final concentration of 1 × 10^6^ cells/mL. Experimental conditions were 20°C with agitation at 130 rpm using orbital shaker IKA KS 4000i control (IKA-Werke, Staufen, Germany). All fermentation vessels were fitted with airlock valves that allowed the release of CO_2_ produced during fermentation while preventing the entry of air and external contaminants. Weight measurements were taken every 24 h to study the accumulation of weight loss. Fermentation was considered to have stopped when the daily weight loss was < 0.1 g/24 h for two consecutive days. In addition, 1 mL of each fermentative product was taken during fermentation every 24 h in order to monitor the metabolic behavior. Bottles were weighed immediately before and after sample collection, which made it possible to exclude the weight of the sample removed from the calculations of cumulative weight loss. Therefore, only the weight loss associated with CO_2_ production was considered during kinetic analysis. At the end of fermentation, cells were separated by centrifugation in a 5810 R centrifuge (Eppendorf, Hamburg, Germany) at 4000 rpm for 6 min. The cell pellet was discarded, and the supernatant was collected and stored at −20 °C until used for analytical determinations.

### 2.4 Kinetic analysis

A kinetic analysis of the fermentative process was carried out for each yeast strain of this study. For this purpose, the accumulation of weight loss during the fermentation was used to calculate the CO_2_ produced at each point of the fermentation and to determine the fermentation profile (Daoud and Searle, 1990). The CO_2_ produced was calculated assuming that the weight lost at each point is lost entirely as CO_2_. The fermentation curve obtained follows the Gompertz equation “Y = A × (exp(− exp ((μ × e)/A) × (λ-x) + 1))” (Zwietering et al., 1990) and the kinetic parameters A (maximum CO_2_ production), μ max (maximum CO_2_ production rate), λ (lag time), and AUC (Area Under the Curve) were showed by fitting the CO_2_ production values to the reparametrised Gompertz equation using GraphPad Prism 8.0.1 software. To characterize the kinetic parameters, OriginPro Version 2025b software (OriginLab Corporation, Northampton, MA, USA) was used. Additionally, the area under the curve (AUC) was calculated using the numerical integration tool (Mathematics > integrate) and using *y* = 0 as the baseline. The results were expressed as the mean and the standard deviation from the independent replicates.

### 2.5 Metabolic analysis

A metabolic analysis of the fermentative process was carried out for each yeast of the initial collection. The milliliter taken from each fermentation each day as well as the final fermentation product were centrifuged to separate the cells from the fermentation product. The extracellular metabolites present in the starting wort and in the fermentative products were analyzed. The main wort sugars (maltotriose, maltose and glucose) and fermentation by-products (ethanol and glycerol) were determined by HPLC (Thermo Fisher Scientific, Waltham, MA), equipped with a HyperREZTM XP Carbohydrate H+ 8 mm column (Thermo Fisher Scientific, Waltham, MA) and a HyperREZTM XP Carbohydrate Guard (Thermo Fisher Scientific, Waltham, MA) using a refraction index detector and a UV/VIS (210 nm) detector. The analysis conditions were: eluent, 5 mM of H_2_SO_4_; 0.6 mL/min flow and oven temperature of 50 °C. The retention times of the eluted peaks were compared with those of the commercial analytical standards (Sigma-Aldrich, Madrid, Spain). Concentrations, in g/L or % (v/v), were quantified from the calibration graphs (R^2^ value > 0.99) of the standards that were previously obtained from a linear curve fit of the peak areas using ten standard mixtures.

### 2.6 Hybrid generation by rare-mating

The generation of hybrids was carried out by the previously described rare-mating procedure with slight modifications (Pérez et al., 2022). The first step was the isolation of natural auxotrophic strains from the two sets of parents (one with strong maltose/maltotriose positive fermentation profile and one with low fermentation profile). For this purpose, the selected parental strains were grown in 10 mL of liquid GPY medium (2% glucose, 0.5% peptone and 0.5% yeast extract) at 28°C under agitation. Then, the cultures were centrifuged and resuspended in 1X PBS for 3 h. After that, cells were recovered and spread on multiple plates with selective media: α-Aminoadipic acid media (α-AA), 5-Fluoorotic acid media (5-FOA) and 5-Fluoroanthranilic acid media (5-FAA). α-Aminoadipic acid media (α-AA) was prepared as described in (Zaret and Sherman, 1985): 2% glucose, 2% agar, 0.17% YNB [-(NH_4_)_2_SO_4_] (Yeast Nitrogen Base free of amino acids and ammonia sulfate, Difco), 30 mg/L Lysine and 2 g/L D,L-α-aminodipate.

5-Fluoroorotic acid media (5-FOA) was prepared as described in (Boeke et al., 1987): 2% glucose, 2% agar, 0.17% YNB [+(NH_4_)_2_SO_4_] (Yeast Nitrogen Base free of amino acids with ammonium sulfate, Difco), 0.1% proline, 0.001% uracil and 1 g/L 5-fluoroorotic acid. 5-Fluoroanthranilic acid media (5-FAA) was prepared as described in (Toyn et al., 2000): 5% glucose, 2% agar, 0.17% YNB [-(NH_4_)_2_SO_4_] (Yeast Nitrogen Base free of amino acids and ammonia sulfate. Difco), an amino acids pool solution (0.01% L-tryptophan, 0.025% adenine, 0.025% uracil, 0.025% L-methionine, 0.025% L-arginine, 0.025% L-histidine, 0.03% L-tyrosine, 0.08% L-leucine, 0.08% L-isoleucine, 0.06% L-lysine, 0.04% L-phenylalanine, 0.15% L-valine, 0.05% L-aspartic acid, 0.2% L-threonine, 0.08% L-glutamic acid, 0.375% L-serine) and 0.5 g/L fluoroanthranilic acid. In the α-AA, 5-FOA and 5-FAA media used, yeasts containing the active version of the *LYS2*, *URA3* and *TRP1* genes, respectively, convert these substances into cytotoxic compounds, inhibiting cell growth. Only those yeasts whose genes are inactive can grow. These auxotrophic yeasts are tested by growth on GPY (positive control), Minimal Medium (negative control, MM: 2% glucose, 2% agar and 0.17% Yeast Nitrogen Base without amino acids) and Minimal Medium supplemented only with the necessary metabolite: lysine, uracil and tryptophan respectively (MM Lys, MM Ura and MM Trp) (Lairón-Peris et al., 2020).

After that, strains carrying the auxotrophic markers were grown separately in 20 mL of GPY for 48 h. Cells were recovered by centrifugation (4000 rpm for 5 min at room temperature) and resuspended in the residual supernatant. Pairs of yeast cultures to be hybridized were placed together, with the condition of having different auxotrophies. Then, 200 μL of each yeast pair mixture was incubated at 25 °C without shaking in 15 mL slant tube containing 2 mL of GPY. After 1, 3, 5, 7 and 10 days, 200 μL of each cross were transferred to MM plates to incubate for no more than 5 days at 28°C. The individual colonies that appeared were isolated individually and a molecular analysis was performed.

### 2.7 Confirmation of hybrid by RFLP analysis

The interspecific *Saccharomyces* hybrid was characterized by PCR amplification of the *GSY1* and *MAG2* nuclear genes and subsequent restriction fragment length polymorphism (RFLP) analysis with *Hinf*I and *Msp*I restriction enzymes, respectively, following the methodology proposed by (González et al., 2008). The analysis of the nuclear genes has been described in (Pérez-Través et al., 2012). The PCR reaction was carried out with NZY Taq II Green Master Mix from NZYtech and specific primers for the *GSY1* (Forward: TTGGAAAAAGAATTTTCGA; Reverse: ATTTCTTGCCACCGGCAAG) and *MAG2* (Forward: TGGTAGAACCGGATATGCA; Reverse: AGCT CCAAGGAATTAGATAC) genes. The PCR programme consisted of 35 cycles: 94 °C for 30 s, 55.5 °C for 30 s and 72 °C for 70 s using the Eppendorf Mastercycler thermocycler (Eppendorf AG, Hamburg, Germany). The PCR product was digested to produce RFLP patterns using FastDigest *Msp*I and *Hinf*I enzymes from ThermoFisher Scientific at the temperature and for the time recommended for each enzyme. The digestion products were resolved on a 3% agarose gel with REALSAFE Nucleic Acid Staining together with two markers: NZYDNA Ladder V (1000 - 100 bp) and 100+50 bp. Comparing the patterns obtained in the digestions for the parents and the hybrid allows to verify the presence of a hybrid strain between the two species.

Five rounds of successive laboratory fermentations were carried out to promote the genetic stabilization of the obtained hybrid yeast, the methodology described in Pérez-Través et al., 2014 with modifications was followed. A single colony of the obtained hybrid was individually inoculated in modified GPY, 1.8% maltose and 0.2% glucose instead of 2% glucose. 10 mL of the modified GPY medium was inoculated and incubated at 20 °C without shaking. After fermentation (7–10 days), an aliquot of 10^7^ cells is used to inoculate a new tube with the same sterile medium and incubated under the same conditions. This process was repeated 5 times.

### 2.8 Fermentative, kinetic and metabolic analyses of parental strains and the hybrid

Fermentations with synthetic wort of the brewing industry described in section “2.2 Beer wort preparation” were carried out for three yeast strains: the two parental strains (Lal 2 and AQ 3504) and the hybrid DR1. 100 mL bottles containing 50 mL of wort were inoculated with starter cultures at a final concentration of 1 × 106 cells/mL. The experimental conditions and fermentation monitoring were carried out as is described in section “2.3 Fermentation experiments.” The kinetic analysis of fermentation is explained in section “2.4 Kinetic analysis” and the metabolic analysis is explained in section “2.5 Metabolic analysis.”

### 2.9 Evaluation of the fermentative capacity of parental and hybrid strains using natural wort

Fermentations with industrial natural wort provided by a brewing company with its original composition without any modification (16.7 ± 0.1 °Bx, pH 5.1) were carried out with the three strains: the two parental strains (Lal 2 and AQ 3504) and the DR1 hybrid. 5 replicates in 1 L bottles containing 800 mL of wort for each strain were inoculated to a final concentration of 1 × 10^6^ cells/mL. Fermentations were carried out at 20°C with agitation at 130 rpm. Bottles were fitted with airlock valves that allowed the release of CO_2_ produced during fermentation while preventing the entry of air and external contaminants. Weight measurements were taken each day to study the accumulation of weight loss. Fermentation was considered to have stopped when the daily weight loss was <1 g/24 h for two consecutive days. At the end of fermentation, 4 L (800 mL for each replicate x 5 replicates) of final product produced by each of the strains under study were recovered and prepared for sensory analysis by carbonation in reusable mini-kegs. The samples were transferred to 4 L kegs and carbonated under controlled CO_2_ pressure using 16 g CO_2_ cartridges and a dispensing system before sensory evaluation.

### 2.10 Analysis of volatile organic metabolites

A metabolic analysis was carried out at the end point for each of the yeast strains using HPLC analysis to determine sugar consumption and alcohol production, as described in section “2.5 Metabolic analysis.” A volatile organic metabolites (VOMs) analysis present in the beer produced by each yeast strain was carried out. VOMs were extracted by headspace solid-phase microextraction (HS-SPME) and they were analyzed by gas chromatography coupled to mass spectrometry (GC–MS).

5 mL samples of each beer sample plus 25 μL of a methanolic solution 1:1000 of 4-fluorobenzaldehyde (internal standard) and 1.5 g of NaCl have been prepared. A 100 μm thick fiber coated with polydimethylsiloxane (Supelco, Bellefonte, USA) was used for the extraction of VOMs. Volatile analysis was performed on an Agilent 7890B gas chromatograph coupled to a 5977B MSD loading an Agilent J&W HP-5MS (5 % phenyl/95 % methylpolysiloxane) capillary column, 30 m × 0.25 mm i.d., 0.25 μm film thickness, using Agilent MassHunter 8.01 SP1 for controlling the analytical platform (Agilent Technologies, Palo Alto, CA, USA). Helium was the carrier gas at a flow rate of 1.6 mL/min and general GC–MS instrumental conditions are summarized in Supplementary Table 1.

Automated GC–MS data processing was performed using freely available Automated Mass Spectral Deconvolution and Identification System (AMDIS) application^1^, as suggested (Cerdán-Calero et al., 2012) and loading an in-house library (Supplementary Table 2) listing specific retention times and ion models of quantified analytes. Analytical settings considered for AMDIS data processing are summarized in Supplementary Table 3.

### 2.11 Sensory analysis of beer produced by DR1 hybrid and its two parental strains

#### 2.11.1 Participants

A total of 102 consumers (59 women, 43 men) took part in the study. The sensory panel consisted of consumers between 18 and 65 years of age (mean ± SD: 35 ± 12.7 years). The age distribution was as follows: 31% aged 18–25 years, 38% aged 26–40 years, and 33% older than 41 years. All were people from the Institute of Agrochemistry and Food Technology (IATA-CSIC) and the University of Valencia. The recruitment criteria to participate in the study was to drink beer at least two or more times in a month.

#### 2.11.2 Procedure

The evaluation of the three samples was monadically following a balanced rotation order (Williams’s design). Participants were provided with a glass of cold beer (approximately 30 ml, coded with three-digit number) and were asked to observe, taste it, and indicate the liking (how much do you like this beer), using a nine-point scale (from “dislike extremely = 1” to “like extremely = 9”) and the purchase intention (would you buy this beer), using a five-point scale (“definitely I would not buy = 1” to “definitely I would buy = 5”). After that, they were asked to taste again the sample and answer to a Check-all-that-apply (CATA) question by checking the attributes that applied to describe the sensory characteristics of each beer from a list of 17 terms. These attributes were: spicy, sweet, mild taste, sour, bitter, floral flavor, orange flavor, pleasant aftertaste, apple taste, fruity taste, alcohol sensation, strong taste, malt/Non-alcoholic beer, rough sensation, atypical taste, body, beer flavor. Participants were instructed to rinse their mouths with water after tasting each sample. The position of the attributes on the screen was balanced across consumers using a Williams Latin square design, while each consumer used the same attribute order to evaluate all samples (Meyners and Castura, 2016). The terms used in the CATA question were obtained from a preliminary session. For that, the samples were presented in pairs, and 10 participants created a list of attributes to describe the similarities and differences between each pair. The participants were told to focus on descriptive parameters such as flavor and texture, avoiding hedonic terms. All terms were annotated by the interviewer, and the most frequently mentioned were selected. Participants responses were collected using a Compusense Cloud (Compusense Inc., Guelph, Canada). Before the commencement of the study, participants were required to sign an informed consent form. The procedure used in this study was approved by the Ethics Committee of the CSIC (Ref: 118/2025).

### 2.12 Data management and statistical analysis

The results obtained from metabolic analyzes by HPLC and GC/MS to obtain concentrations of sugars, alcohols, and VOMs were analyzed and represented as bar graphs using GraphPad Prism 8.0.1 software. The results obtained from the three biological replicates (for fermentations in synthetic wort representative of the brewing industry) and for the five biological replicates (for commercial fermentations in industrial wort) were represented as the mean with the standard deviation (SD). Differences among parental strains and the DR1 hybrid were evaluated using an ordinary one-way analysis of variance (ANOVA) followed by Tukey’s honestly significant difference (HSD) post-hoc test for multiple comparisons (*p* < 0.05), which is suitable and robust for continuous analytical data across balanced groups.

The values obtained from liking and purchase intention were analyzed using a mix-model analysis of variance (ANOVA) considering products as a fixed effect and participants as a random effect. No additional covariates were included. Residuals were inspected graphically to verify normality and homogeneity of variance assumptions prior to inference. Tukey’s test was used for a *post hoc* comparison of the means among samples to determine significant differences (*p* < 0.05). For the CATA question, for each beer, the number of times that each attribute was selected by participants was counted. Then, the Cochran’s Q test was carried in order to identify significant differences between the samples for each CATA term. Subsequently, a penalty analysis of the CATA data was performed for overall liking, to determine the drop-in liking associated with the presence or absence of each attribute from the CATA question. Statistical analyses of sensory data were performed using XLSTAT statistical software (version 2021, Addinsoft, Paris, France).

In order to correlate the VOMs analysis and the sensory analysis, a Principal Component Analysis (PCA) was carried out using OriginPro Version 2025b software (OriginLab Corporation, Northampton, MA, USA) was used. The Odor Activity Value (OAV) was calculated dividing the concentration of each VOM by the reported odor threshold for beer. The OAV data and the percentages for each attribute in the sensory analysis were used to create the correlation matrix, which was represented by a biplot, relating each of the strains (sample scores) to the aromas/sensory attributes (loadings). All variables were autoscaled before PCA.

## 3 Results

### 3.1 Selection of parental strains for hybridization

The aim of this project was to generate new yeast hybrids with lower ethanol production and enhanced aroma profile without losing fermentative power by using rare-mating techniques and to perform an analytical and sensory analysis of the beer produced by these hybrids. A collection of 28 yeast strains was analyzed during beer fermentation through kinetic and metabolic analyses. Fermentation kinetics were assessed by monitoring weight loss, allowing the determination of CO_2_ production, the area under the curve (AUC), and the kinetic parameters A (maximum CO_2_ production), μ max (maximum CO_2_ production rate) and λ (lag phase) (Table 1). Metabolic analysis determined the consumption of the main wort sugars (glucose, maltose and maltotriose) and the production of ethanol and glycerol. The combination of both analyses enabled the selection of strains of interest as parents for hybrid generation (Figures 1, 2).

**FIGURE 1:**
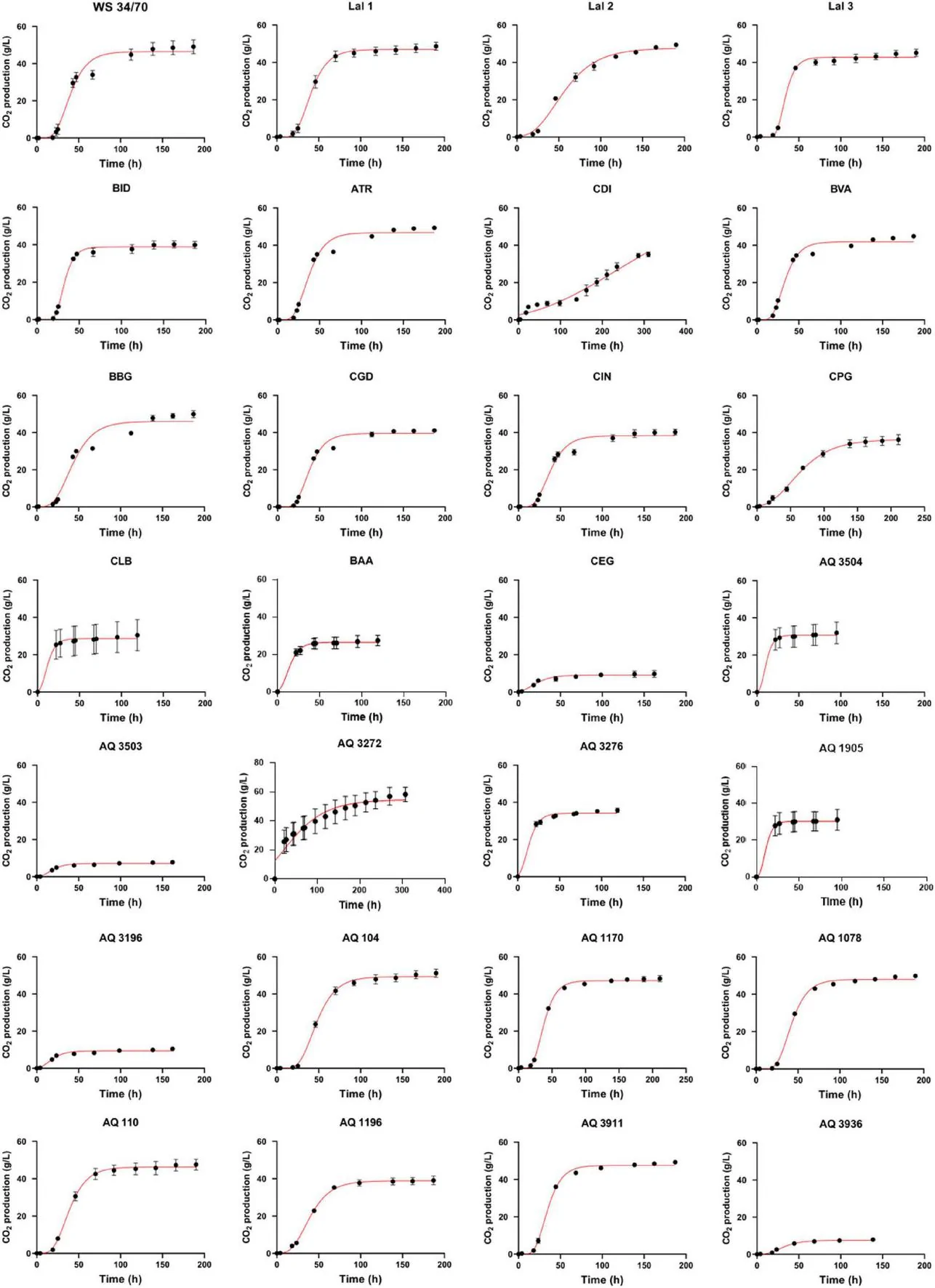
Kinetic analysis of the fermentation performed by the 28 yeast strains candidates as parental for hybrid generation in brewing conditions by fitting the CO_2_ produced at each point to the reparametrised Gompertz equation.

**FIGURE 2:**
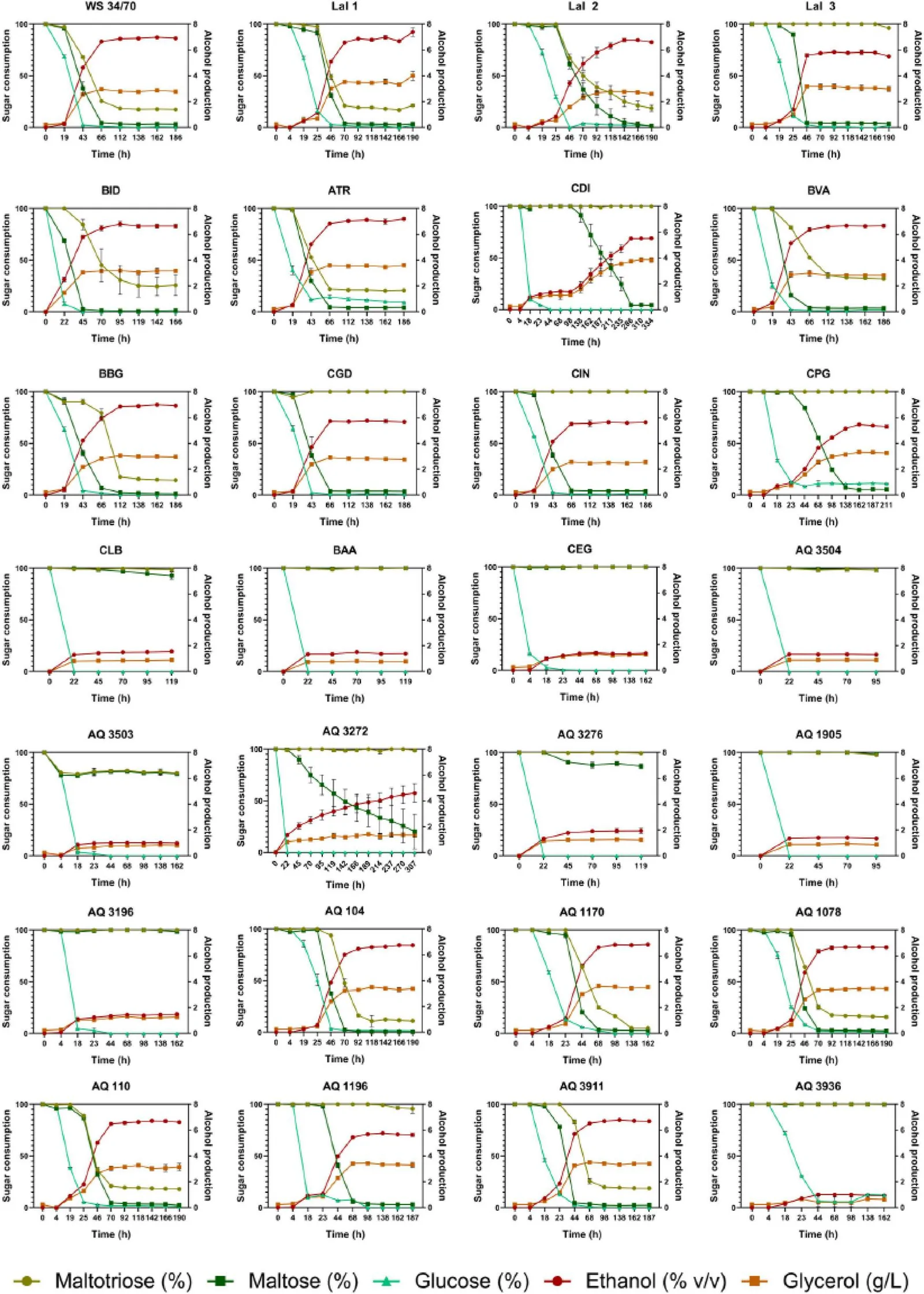
Metabolic analysis for the 28 yeast strains candidates as parental for hybrid generation in brewing conditions by representing the maltotriose (%), maltose (%) and glucose (%). consumption and ethanol (% v/v) and glycerol (g/L) production at each point of the fermentation.

We first analyzed the fermentation parameters of the different strains. Yeasts belonging to *S. cerevisiae* or *S. cerevisiae* hybrids, related to fermentation in brewing environments, show a higher “A” parameter, as AQ 104 (4.93 ± 0.22 × 10^1^ g/L) or Lal 2 (4.77 ± 0.04 × 10^1^ g/L). Other yeast strains from brewing environments, such as AQ 1078 or AQ 1170 (*S. cerevisiae* x *S. kudriavzevii*) exhibit kinetic parameters that indicate they are well adapted to alcoholic fermentation in this type of wort medium.

Daily monitoring of weight loss allowed the recording of the actual fermentation kinetics of each yeast strain in triplicate and the calculation of the area under the curve (AUC; mean ± standard deviation). The fermentation profiles revealed differences in both rate and extent of fermentation among strains. Metabolic analysis by HPLC quantified the consumption of major sugars (glucose, maltose, and maltotriose) and the production of ethanol and glycerol at distinct fermentation stages and at the endpoint. Depending on maltose (Mt) or maltotriose (Mtt) consumption and considering that they are all able to metabolize glucose, three types of profiles have been obtained: Mtt−/Mt−, Mtt−/Mt+, and Mtt+/Mt+. Yeasts such as WS 34/70, Lal 1, Lal 2, BID, AQ 104, AQ 1170, AQ 1078 or AQ 110 show metabolic behavior typical of brewing yeasts, there is a total maltose consumption and a very high maltotriose consumption, leading to ethanol yields of 6–8 % (v/v). BVA, BBG or AQ 3911 show a lower or later maltotriose uptake, they are strains whose adaptation to complex sugar uptake is slower or less efficient. Lal 3 is an industrial yeast that does not consume maltotriose. Similarly, strains CDI, CGD, CIN and CPG do not consume maltotriose but do consume maltose. Finally, there are yeasts unable to consume maltotriose or maltose such as CLB, BAA, CEG, AQ 3504, AQ 3503, AQ 3276, AQ 1905, AQ 3196 or AQ 3936. No yeast strains that consume maltotriose but not maltose (Mtt+/Mt−) have been found.

The selection of parents was based on the analysis of kinetic parameters, sugar consumption, and ethanol and glycerol production. On the one hand, we selected strains with a good fermentation capacity and complete and efficient sugar consumption, with the aim of providing the hybrid a fermentation profile typical of the brewing industry. On the other hand, parents have been selected with a reduced ethanol production. For the first objective, the following have been selected: strains WS 34/70, Lal 1 and Lal 2, because of their commercial use, good maltose and maltotriose consumption and good kinetic parameters or AUC. In addition, strain BBG, a strain from the Ale beer subpopulation, which is slower to adapt to maltotriose consumption, was selected.

For the second objective, two strains of *S. arboricola*, AQ 1905 and AQ 3196, have been selected: these strains show zero maltotriose and maltose consumption, resulting in minimal ethanol production, under 2 % (v/v). In addition, *S. paradoxus* strain AQ 3504 was selected, which does not consume maltose and maltotriose but shows kinetic parameters that reflect good adaptation to brewing conditions, it shows one of the highest μ max values (2.09 ± 0.43 h_–1_) and a low λ value (3.56 ± 0.13 h). AQ 3936 of the genus *Kazachstania* has also been used as a representative of non-*Saccharomyces* yeasts.

### 3.2 Generation and confirmation of hybrid

The first step in generating hybrids by rare mating was the construction and verification of a collection of auxotrophic derivatives from the selected parental strains. Auxotrophs (Lys^–^, Ura^–^ and Trp^–^) were isolated using selective α-AA, 5-FOA and 5-FAA media and subsequently confirmed on supplemented minimal media. The verified auxotrophic strains were then used as parental strains for hybrid generation (Supplementary Table 4). Nine strains were selected as candidates for auxotrophic strains generation. Auxotrophic strains were successfully isolated from AQ 3196 (7 Lys^–^, 4 Ura^–^ and 2 Trp^–^), AQ 3504 (6 Lys^–^ and 2 Trp^–^) and Lal 2 (1 Lys^–^), while no auxotrophic mutants were obtained from WS 34/70, Lal 1, BBG, CGD, AQ 1905 or AQ 3936 (Supplementary Table 4).

One auxotroph was obtained for the strains considered as strong fermenters, a Lys^–^ strain of Lal 2. In contrast, auxotrophs were obtained for AQ 3196 (*S. arboricola*) and AQ 3504 (*S. paradoxus*) for the lowest sugar-consuming strains. By crossing different auxotrophic strains and testing in minimal medium, a hybrid between *S. cerevisiae* (Lal 2) and *S. paradoxus* (AQ 3504) was obtained, named as DR1.

The characterization of the interspecific hybrid DR1 was carried out using PCR-RFLP analysis by amplifying the *GSY1* and *MAG2* genes for subsequent digestion with the Hinf I and Msp I enzymes, respectively.

The RFLP pattern for the *GSY1* and *MAG2* genes when digested by Hinf I and Msp I allows verification of the existence of the DR1 hybrid generated from the parental strains (P1 and P2; *S. cerevisiae* and *S. paradoxus*) (Figure 3). The *GSY1* pattern when digested with Hinf I shows a 240 bp band in *S. paradoxus* that is not found in the *S. cerevisiae* pattern. The pattern for the DR1 hybrid (*S.c* x *S.p*) shows a combination of both patterns. On the other hand, the *MAG2* pattern when digested by Msp I shows two bands over 200 bp, 490 and 400 bp, for *S. cerevisiae*, while it shows two bands over 200 bp, 350 and 225 bp, for *S. paradoxus*. The pattern for the DR1 hybrid (S.c x S.p) shows the combination of both patterns, showing 4 bands over 200 bp: 490, 400, 350 and 225 bp.

**FIGURE 3:**
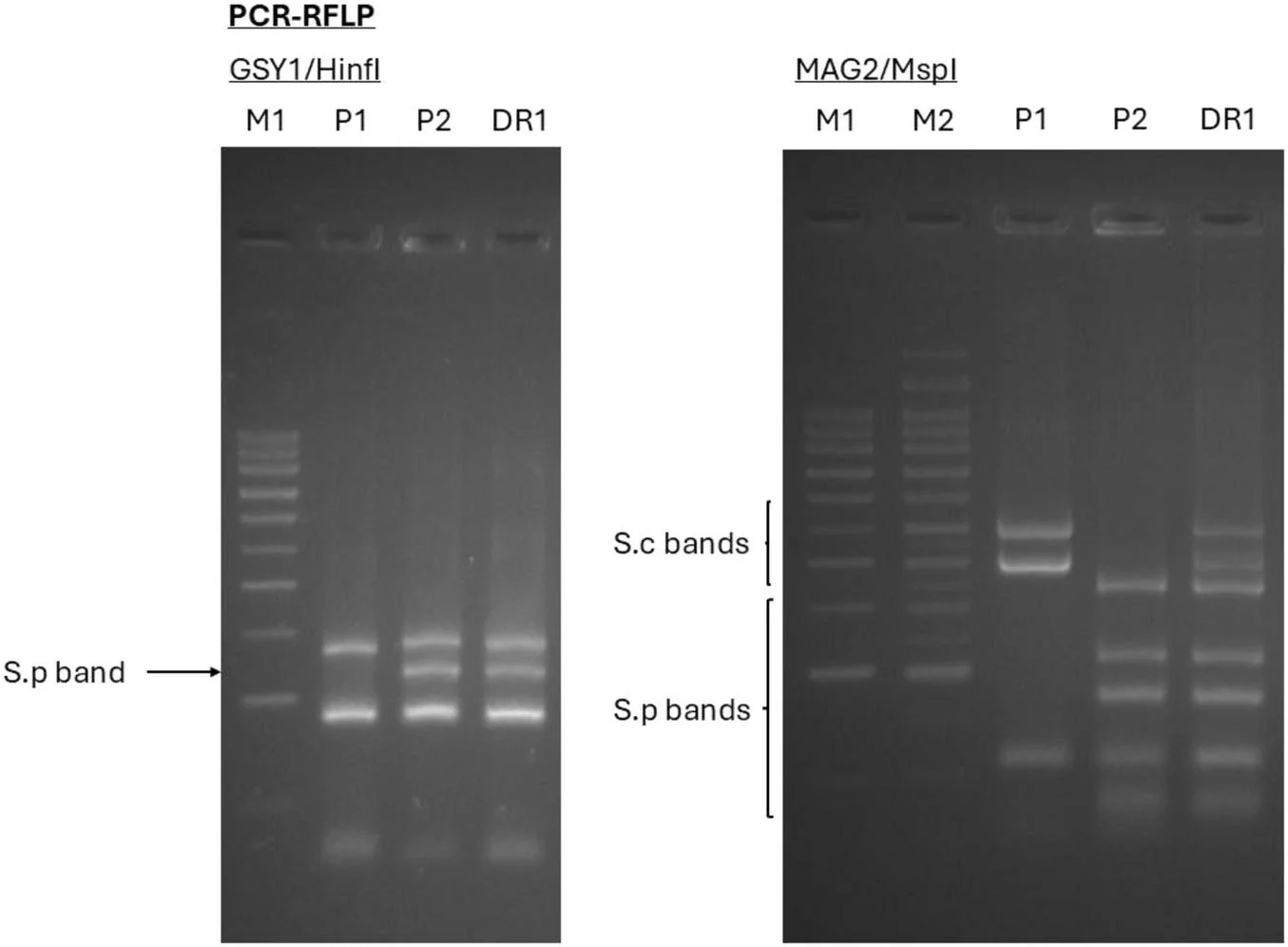
Genetic characterisation of the interspecific hybrid DR1 by PCR-RFLP. *GSY* gene was digested by HinfI and *MAG2* gene was digested by MspI. The patterns obtained for DR1 are combination of *S. cerevisiae* and *S. paradoxus* bands. M1: molecular marker 1, NZY DNA Ladder V. M2: molecular marker 2, mix of 100 bp and 50 bp DNA Ladder. P1: parental strain 1, Lal 2 strain (*S. cerevisiae*). P2: parental strain 2, AQ 3504 (*S. paradoxus*). DR1: hybrid strain DR1 (*S. cerevisiae* x *S. paradoxus*).

### 3.3 Analysis of the fermentative capacity of the DR1 hybrid strain

Fermentations were carried out with synthetic wort representative of the brewing industry using the parental strains (Lal 2, *S. cerevisiae*, and AQ 3504, *S. paradoxus*) and the hybrid DR1 (*S. cerevisiae* x *S. paradoxus*). The kinetic and metabolic behavior of the strains was analyzed by monitoring fermentation and analyzing the final product by HPLC. Monitoring weight loss under fermentation conditions made it possible to determine the concentration of CO_2_ produced at each point and the kinetic parameters of the Gompertz equation (Table 2).

**TABLE 2:** Kinetic parameters of the reparametrized Gompertz equation for the DR1 hybrid (*S. cerevisiae* x *S. paradoxus*) and the parental strains Lal 2 (*S. cerevisiae*) and AQ 3504 (*S. paradoxus*) in brewing conditions with synthetic brewing wort.

Yeast strain	AUC	A (g/L)	μ max (h_–1_)	λ (h)
Lal 2 (*S.c*)	6.57 ± 0.52 x 10^3^	5.06 ± 0.23 x 10^1^	0.96 ± 0.24	2.47 ± 0.26 x 10^1^
AQ 3504 (*S.p*)	1.67 ± 0.13 x 10^3^	1.08 ± 0.07 x 10^1^	0.26 ± 0.08	5.97 ± 3.39
DR1 (*S.c* x *S.p*)	5.78 ± 0.39 x 10^3^	4.24 ± 0.13 x 10^1^	0.82 ± 0.14	1.99 ± 0.15 x 10^1^

The values show the mean of the replicates (*n* = 3) and their standard deviation (SD). Area under the curve (AUC), asymptote with 0 CO_2_ production (A) as maximum CO_2_ produced (g/L), maximum CO_2_ production rate (μ max) (h_–1_) and lag time (λ) (h) are shown.

The maximum CO_2_ production (parameter “A”) shows a higher value in the Lal 2 strain (5.06 ± 0.23 × 10^1^ g/L). It was selected as a parental strain due to its good fermentative capacity. The DR1 hybrid also shows a high maximum CO_2_ production (4.24 ± 0.13 × 10^1^g/L). Similarly, the values of the parameters “μ” and “λ” show their highest values in the commercial strain for beer production Lal 2 (0.96 ± 0.24 h^–1^ and 2.47 ± 0.26 × 10^1^h), while the DR1 hybrid also shows high values (0.82 ± 0.14 h^–1^ and 1.99 ± 0.15 × 10^1^ h), closer to the commercial parental strain Lal 2 than to the wild parental strain AQ3504.

The metabolic behavior was analyzed by HPLC analysis of the final fermentation product in order to determine the consumption of the main sugars present in brewing wort (maltotriose, maltose and glucose) and the production of ethanol and glycerol by the yeast strains (Supplementary Table 5).

Lal 2 (*S. cerevisiae*) shows almost complete consumption of all the main sugars (Figure 4). It is able to metabolize 100 % of maltose and glucose, and 91.22 ± 1.38 % of maltotriose. On the other hand, AQ 3504 (*S. paradoxus*) shows poor consumption of the main sugars, being able to metabolize only glucose. The DR1 hybrid (*S.c* x *S.p*) is able to metabolize 100 % of glucose like both parents and 96.26 ± 0.23 % of maltose like the commercial parental strain (Lal 2). However, it does not have the ability to metabolize maltotriose like the wild parental strain (AQ 3504). Lal 2, as a commercial strain, achieves 6.57 ± 0.05 % v/v ethanol production. DR1, due to its good consumption of the predominant sugar, maltose, produces 5.20 ± 0.10 % v/v ethanol. This results in a significant reduction (21 %) in the alcohol content of the final product compared to the commercial parental strain. However, this is not the same situation with glycerol production, which is higher in the DR1 hybrid than in the Lal 2 parental strain. DR1 produces 2.69 ± 0.69 g/L of glycerol, while the Lal 2 strain produces 2.26 ± 0.36 g/L.

**FIGURE 4:**
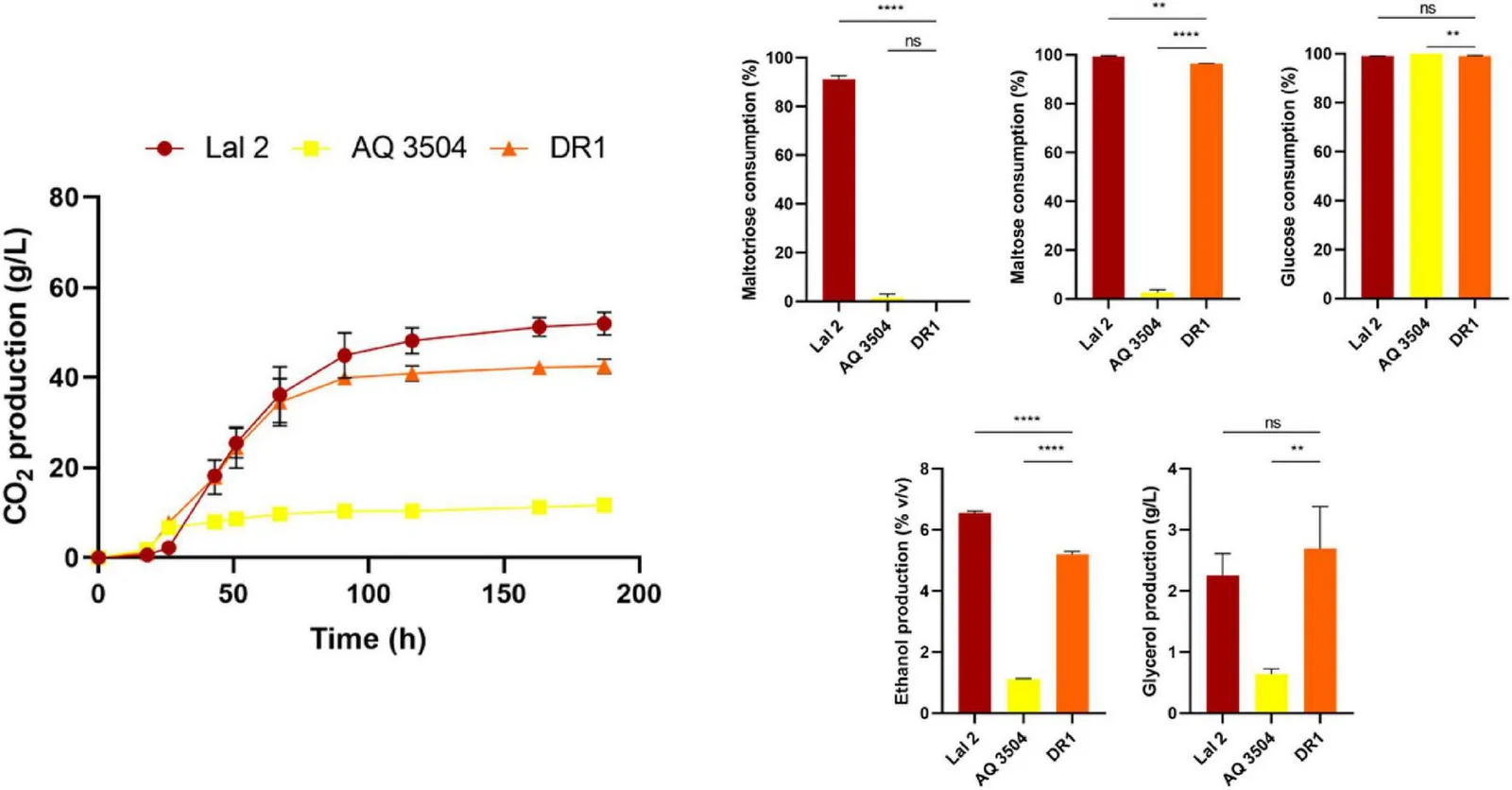
Metabolic analysis of the DR1 interspecific hybrid (*S. cerevisiae* x *S. paradoxus*) and the parental strains Lal 2 (*S. cerevisiae*) and AQ 3504 (*S. paradoxus*) in brewing conditions with synthetic brewing wort. The CO_2_ production (g/L) is calculated based on weight loss during fermentation and represents the progression of fermentation. Endpoint metabolic analysis of the three strains is performed in order to compare the parental strains with the DR1 hybrid. Maltotriose (%), maltose (%) and glucose (%) consumption and ethanol (% v/v) and glycerol (g/L) production is shown. Asterisks (*) indicate significant differences according to ANOVA test with multiple comparisons, Tukey’s HSD test (*p < 0.05, **p < 0.01, ***p < 0.001, ****p < 0.0001).

### 3.4 Kinetic and metabolic analysis of the DR1 hybrid in industrial conditions

Fermentations with the parental strains (Lal 2, *S. cerevisiae*, and AQ 3504, *S. paradoxus*) and the hybrid DR1 (*S. cerevisiae* x *S. paradoxus*) were repeated using an industrial wort (16.7 ± 0.1 °Bx, pH 5.1, corresponding to an initial concentration of maltose of 74.36 ± 0.56 g/L and an initial concentration of maltotriose of 28.88 ± 0.03 g/L). Under these simulated industrial conditions due to industrial wort utilization, five biological replicates have been used, and the volume was increased to 800 mL. Kinetic and metabolic analysis were performed by monitoring the fermentation progress and analyzing the final product by HPLC. Monitoring weight loss under fermentation conditions made it possible to determine the concentration of CO_2_ produced at each point and the kinetic fermentative parameters (Table 3).

**TABLE 3:** Kinetic parameters of fermentation for the DR1 interspecific hybrid (*S. cerevisiae* x *S. paradoxus*) and the parental strains Lal 2 (*S. cerevisiae*) and AQ 3504 (*S. paradoxus*) in real brewing conditions with industrial brewing wort.

Yeast strain	AUC	A (g/L)	μ max (h_–1_)	λ (h)
Lal 2 (*S.c*)	1.70 ± 0.05 x 10^4^	5.84 ± 0.16 x 10^1^	0.32 ± 0.01	0.38 ± 1.79
AQ 3504 (*S.p*)	3.56 ± 0.25 x 10^3^	9.42 ± 0.86	0.40 ± 0.17	8.70 ± 4.41
DR1 (*S.c* x *S.p*)	1.21 ± 0.07 x 10^4^	5.00 ± 0.20 x 10^1^	0.18 ± 0.03	2.17 ± 5.25

The values show the mean of the replicates (*n* = 5) and their standard deviation (SD). Area under the curve (AUC), (A) maximum CO_2_ produced (g/L), maximum CO_2_ production (μ max) (h_–1_) and lag time (λ) (h) are shown.

The maximum CO_2_ production (parameter “A”) shows again its highest value in the Lal 2 strain (5.84 ± 0.16 × 10^1^ g/L) due to be the commercial one. DR1 hybrid shows again a high A value (5.00 ± 0.20 × 10^1^ g/L). This suggest that good fermentation capacity was inherited from the commercial strain. Moreover, the “λ” parameter, which represents the strain’s latency, shows its highest value in strain AQ 3504 (8.70 ± 4.41 h), whereas is lowest for Lal 2, which means that DR1 hybrid has a latency that lies between those of its parents but is closer to the latency of Lal 2. In the case of the parameter “μ,” DR1 has a lower CO_2_ production rate than its parents. Finally, AUC obtained for the DR1 hybrid (1.21 ± 0.07 × 10^4^) is closer to that obtained in Lal 2 (1.70 ± 0.05 × 10^4^) than in AQ 3504 (3.56 ± 0.25 × 10^3^).

The metabolic behavior was analyzed again by HPLC analysis of the final fermentation product in order to determine the consumption of the main sugars present in brewing wort (maltotriose, maltose and glucose) and the production of ethanol and glycerol by the yeast strains (Supplementary Table 6).

The parental strains selected for hybrid generation show distinct metabolic profiles, while still sharing certain common features (Figure 5). The two parental strains, like the DR1 hybrid, show complete glucose consumption. Lal 2 (*S. cerevisiae*), as most industrial brewing strains, it shows almost complete consumption of main sugars, maltose (98.21 ± 0.45 %, corresponding to a residual maltose concentration of 1.32 ± 0.33 g/L) and maltotriose (86.77 ± 1.10 %, corresponding to a residual maltotriose concentration of 3.82 ± 0.32 g/L). Something very different occurs in AQ 3504 (*S. paradoxus*), whose consumption of main sugars is practically non-existent, maltose (1.61 ± 2.85 %, corresponding to a residual maltose concentration of 73.16 ± 2.12 g/L) and maltotriose (2.53 ± 2.87 %, corresponding to a residual maltotriose concentration of 28.16 ± 0.83 g/L). The DR1 hybrid shows an intermediate phenotype between the two parental strains. It has adopted the ability to consume maltose (96.85 ± 0.06 %, corresponding to a residual maltose concentration of 2.34 ± 0.04 g/L) from the commercial parental strain but is not able to consume maltotriose (7.47 ± 0.83 %, corresponding to a residual maltotriose concentration of 26.73 ± 0.24 g/L) like the wild parental strain. The residual maltotriose concentration of DR1 contributes to body and sweetness but may also increase susceptibility to spoilage by microorganisms capable of utilizing this sugar, highlighting the need for dedicated shelf-life and microbiological stability studies prior to industrial application. As for alcohol production, ethanol production is high in the commercial parent and low in the wild parent. The DR1 hybrid shows intermediate production with a significant difference from both parents, producing 6.21 ± 0.10 % v/v, which is a moderate decrease (17.5 %) compared to Lal 2 (7.53 ± 0.10 % v/v). However, maximum glycerol production was found in the DR1 hybrid (3.46 ± 0.40 g/L) compared to Lal 2 (2.33 ± 0.26 g/L) and AQ 3504 (1.50 ± 0.05 g/L).

**FIGURE 5:**
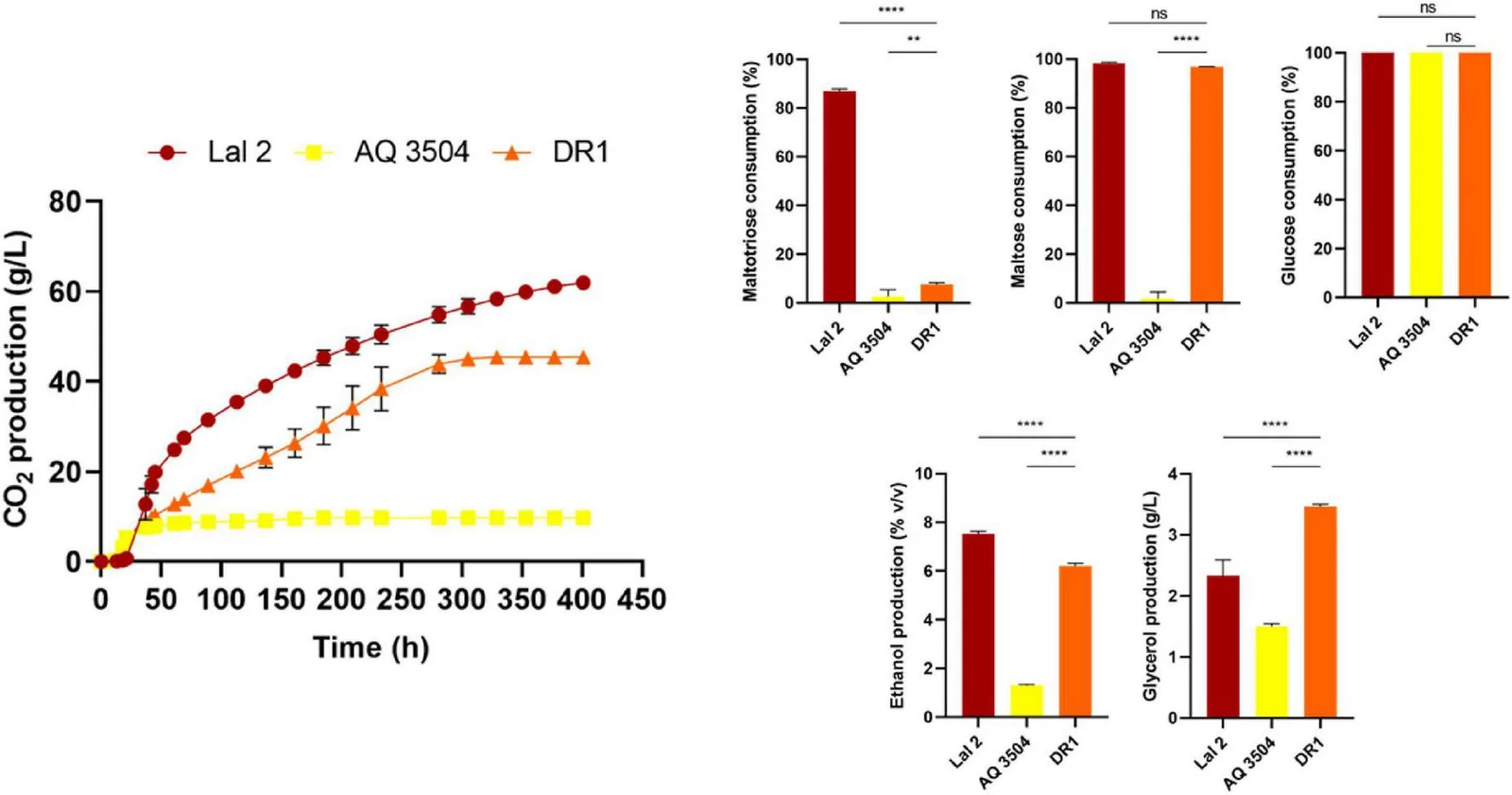
Metabolic analysis of the DR1 hybrid (*S. cerevisiae* x *S. paradoxus*) and the parental strains Lal 2 (*S. cerevisiae*) and AQ 3504 (*S. paradoxus*) in industrial brewing conditions with industrial brewing wort. The CO_2_ production (g/L) is calculated based on weight loss during fermentation and represents the progression of fermentation. Endpoint metabolic analysis of the three strains is performed in order to compare the parental strains with the DR1 hybrid. Maltotriose (%), maltose (%) and glucose (%) consumption and ethanol (% v/v) and glycerol (g/L) production is shown. Asterisks (*) indicate significant differences according to ANOVA test with multiple comparisons, Tukey’s HSD test (*p < 0.05, **p < 0.01, ***p < 0.001, ****p < 0.0001).

### 3.5 Volatile organic metabolites analysis of the DR1 hybrid in industrial conditions

The aroma profile under brewing conditions of the two parental strains and the DR1 hybrid was analyzed by GC/MS analysis of Volatile Organic Metabolites (VOMs). A total of 38 volatile compounds were detected, with semi-quantification performed relative to the internal standard. These compounds belong mainly to the functional groups: higher alcohol, aldehydes, acids, acetate esters and fatty acid esters (Figure 6).

**FIGURE 6:**
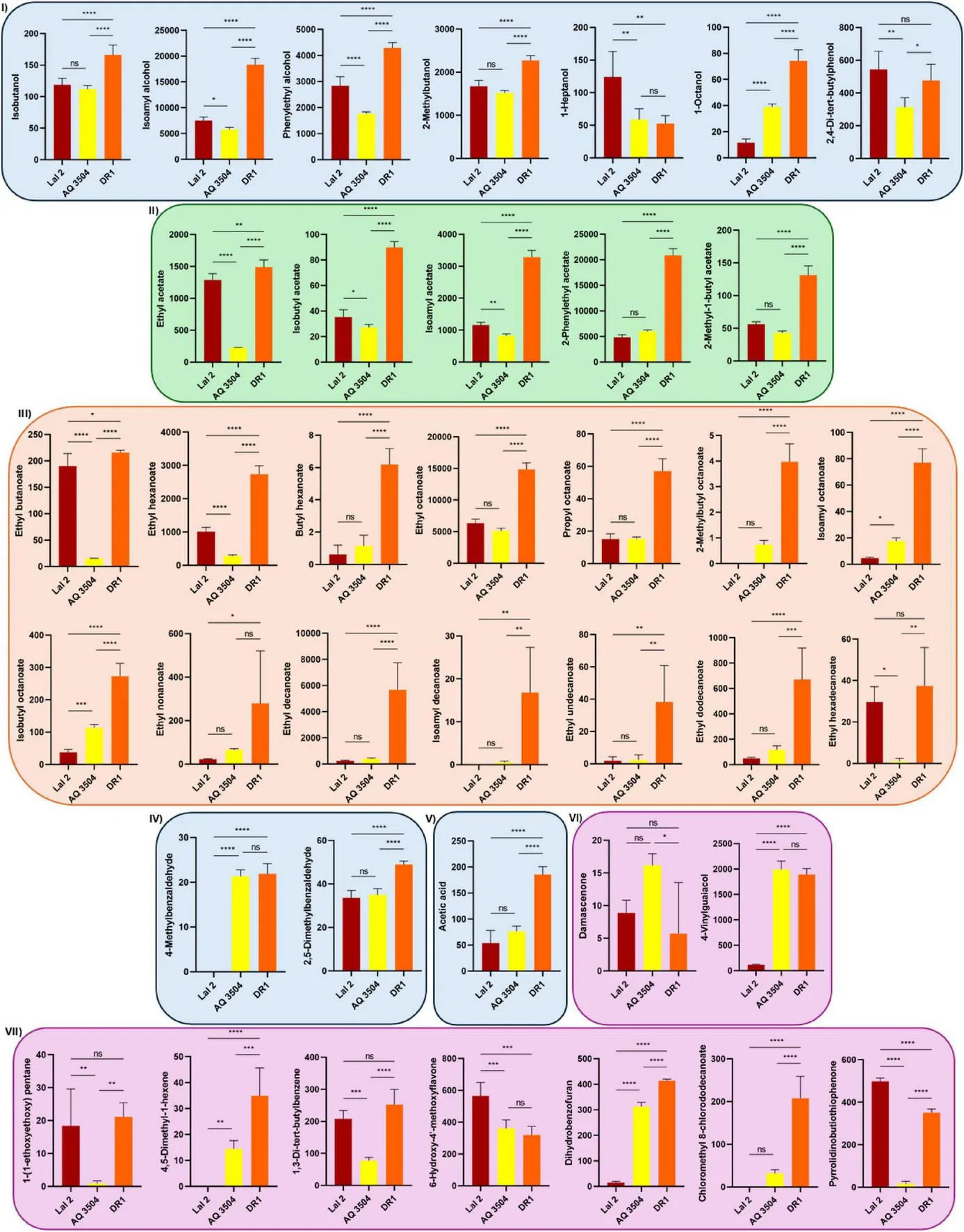
Final concentrations (μg/L) of volatile organic metabolites produced by the DR1 hybrid (*S. cerevisiae* × *S. paradoxus*) and its parental strains, Lal 2 (*S. cerevisiae*) and AQ 3504 (*S. paradoxus*), during fermentation of industrial brewing wort. Compounds are organized into the following chemical groups: I, higher alcohols; II, acetate esters; III, fatty acid esters; IV, aldehydes; V, acids; VI, brewing-relevant compounds; and VII, other compounds. Values represent the mean ± standard deviation of five independent biological replicates. Differences among strains were evaluated separately for each compound using one-way ANOVA followed by Tukey’s HSD test. Asterisks indicate significant differences: *p* < 0.05, *p* < 0.01, *p* < 0.001, and *p* < 0.0001. Numerical concentrations, beer perception thresholds, odor activity values, odor descriptors, and references for compounds with established thresholds are provided in Table 4.

The higher alcohols that have been analyzed in this study are isobutanol, isoamyl alcohol, phenylethyl alcohol, 2-methylbutanol, 1-heptanol, 1-octanol and 2,4-Di-tert-butylphenol. These 7 higher alcohols are generally produced in higher concentrations by the commercial parental strain (Lal 2) than by the wild parental strain (AQ 3504), with the exception of 1-octanol, which is produced in higher concentrations by AQ 3504 (3.90 ± 0.23 × 10^1^ μg/L). The DR1 hybrid shows higher production of higher alcohols than both parents in practically all quantified higher alcohols in this study. Remarkable is the high production of isoamyl alcohol (1.84 ± 0.12 × 10^4^ μg/L) and 1-octanol (7.43 ± 0.85 × 10^1^ μg/L), exceeding two times the production of Lal 2 and AQ 3504, respectively. However, the production of 1-heptanol is low in both the DR1 hybrid and AQ 3504, being exceeded by that produced in Lal 2 (1.07 ± 0.50 × 10^2^ μg/L).

The two aldehydes quantified are 4-methylbenzaldehyde and 2,5-dimethylbenzaldehyde. The first is not produced by Lal 2, and the second was quantified at a higher concentration in the DR1 hybrid than in the parents (4.89 ± 0.15 × 10^1^ μg/L). The only 1 acid detected in this study was acetic acid, which was found at a very high concentration in DR1 (1.85 ± 0.15 × 10^2^ μg/L).

The five acetate esters that have been analyzed in this study are ethyl acetate, isobutyl acetate, isoamyl acetate, 2-phenylethyl acetate and 2-methyl-1-butyl acetate. In general, the DR1 hybrid shows an overproduction of acetate esters. The production of isoamyl acetate (3.29 ± 0.21 × 10^3^ μg/L) and 2-phenylethyl acetate (2.08 ± 0.13 × 10^4^ μg/L) are 3 and 4 times higher than that produced by the parental strains. 14 fatty acid esters were detected, some of them of great aromatic importance, such as ethyl butanoate, ethyl hexanoate, butyl hexanoate, ethyl octanoate, isoamyl octanoate or isoamyl decanoate. Again, the DR1 hybrid shows an overproduction of fatty acid esters. These compounds are produced by the DR1 hybrid in a much higher concentration than in the parental strains. The production of ethyl hexanoate (2.73 ± 0.26 × 10^3^ μg/L), butyl hexanoate (6.18 ± 0.98 μg/L) or ethyl octanoate (1.48 ± 0.11 × 10^4^ μg/L) is much higher than in the parents.

Two compounds of high interest for the brewing industry were identified and quantified: damascenone and 4-vinylguaiacol. The wild parental strain (1.62 ± 0.18 × 10^1^ μg/L) has a higher damascenone production than that found in the DR1 hybrid (5.71 ± 7.82 μg/L). The production of 4-vinylguaiacol is minimal in the fermentation of Lal 2 (1.11 ± 0.14 × 10^2^ μg/L).

The other seven compounds are very varied. It can be seen that the DR1 hybrid has similar production levels of these compounds to one of the two parents, with high production of 1-(1-ethoxyethoxy)pentane (21.10 ± 4.31 μg/L) or 1,3-Di-tert-butylbenzene (2.52 ± 0.48 × 10^2^ μg/L) as Lal 2, or a lower production of 6-hydroxy-4′-methoxyflavone (3.20 ± 0.55 × 10^2^ μg/L) as AQ 3504. However, there are also compounds where the DR1 hybrid outperforms both parents, such as 4,5-dimethyl-1-hexene (3.50 ± 1.07 × 10^1^ μg/L), a compound that is not detected in Lal 2, or dihydrobenzofuran (4.13 ± 0.07 × 10^2^ μg/L), a compound that has a very low production in Lal 2.

### 3.6 Sensory profile, acceptance and purchase intention of the beer

The results of the analysis are presented in Figure 7, which shows the frequency with which each attribute was selected to describe each beer. Of the 17 terms listed in the CATA question, 15 presented significant differences between samples (except, floral and orange flavor) according to the non-parametric Cochran’s Q test. These results indicated that, in general, the consumer perceived differences among samples. Overall, the sensory profile of Lal 2 and DR1 was quite similar. The first beer was described principally as bitter, with beer flavor, and strong taste, and also it presents alcohol sensation. Similarly, DR1 was described as bitter, with beer and strong taste, and also with alcohol sensation, but it was described with an atypical taste. The sensory profile of AQ 3504 was different of the other two beers. This was described as sweet, with fruity taste, bitter and mild taste, principally, but participants also described them with an atypical flavor.

**FIGURE 7:**
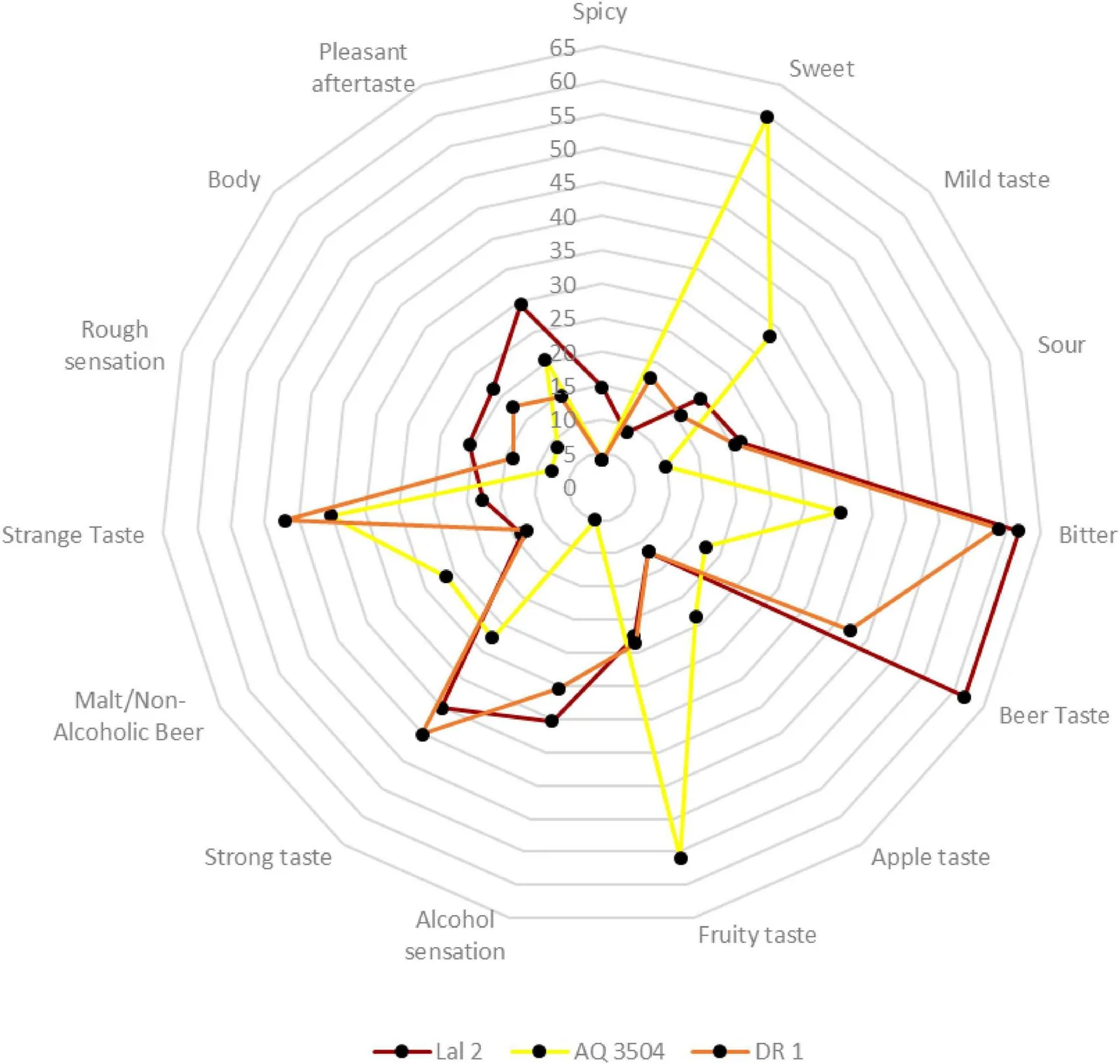
Sensory profile of the three beers. Only the terms where we observed significant differences *p* < 0.05 are shown accordingly to Cochran’s Q test.

Concerning liking, ANOVA results indicated that liking scores varied significantly among samples (*p* < 0.05). The mean values ranging from 4.7 to 5.6, being the highest score for Lal 2 beer and the lowest for AQ 3504 and DR1. The latter two samples were similar to each other, but significantly different to Lal 2 (*p* < 0.05) according to the Tukey test. Similarly, results indicated a higher purchase intention for Lal 2 beer (mean value = 3.0) than for AQ 3504 and DR1 (2.6 and 2.5, respectively). To determine which attributes had the greatest impact on liking, penalty analysis was performed. The results showed that pleasant aftertaste, beer flavor, body, mild taste, fruity taste, and alcohol sensation increased liking, whereas atypical taste, rough sensation, and strong taste had a negative impact and decreased overall liking.

### 3.7 Odour activity values and relationship with sensory perception

Odor Activity Values (OAVs) were calculated to evaluate the sensory relevance of VOMs. They were calculated by dividing the concentration of each VOM by its reported odor threshold in beer matrix (Table 4). Although numerous VOMs were detected, only a limited number reached the threshold value, reporting a OAV above 1, and indicating a likely direct contribution to beer aroma.

**TABLE 4:** Concentration (μg/L), odor thresholds (μg/L), odor activity values (OAVs), and odor descriptors of aroma-active volatile compounds identified in beers produced by Lal 2, AQ 3504 and DR1 strains.

Volatile organic metabolite (μg/L)	Threshold (μg/L)	Concentration in Lal 2 beer (μg/L)	Concentration in AQ 3504 beer (μg/L)	Concentration in DR1 beer (μg/L)	Lal 2 (OAV)	AQ 3504 (OAV)	DR1 (OAV)	Odor description	References
Isobutanol	200000	118.44 ± 10.65	111.81 ± 5.70	166.33 ± 15.50	0.00 ± 0.00	0.00 ± 0.00	0.00 ± 0.00	Alcohol, solvent, fusel	Meilgaard, 1975
Isoamyl alcohol	70000	7526.76 ± 690.05	5807.43 ± 397.50	18386.82 ± 1181.75	0.11 ± 0.01	0.08 ± 0.01	0.26 ± 0.02	Alcohol, banana, sweetish, aromatic	
2-Methylbutanol	65000	1671.28 ± 140.87	1518.11 ± 56.06	2276.84 ± 106.16	0.03 ± 0.00	0.02 ± 0.00	0.04 ± 0.00	Alcohol, banana, medicinal, solvent	
1-Heptanol	1000	107.38 ± 50.42	58.91 ± 16.56	42.39 ± 25.87	0.11 ± 0.05	0.06 ± 0.02	0.04 ± 0.03	Coconut, unpleasant, ketonic solvent	
1-Octanol	900	11.74 ± 2.79	38.99 ± 2.32	74.28 ± 8.46	0.01 ± 0.00	0.04 ± 0.00	0.08 ± 0.01	Coconut, Walnut, oily	
Phenylethyl alcohol	125000	2833.50 ± 356.02	1759.95 ± 73.28	4290.50 ± 199.56	0.02 ± 0.00	0.01 ± 0.00	0.03 ± 0.00	Roses, floral, sweetish, perfumed	Holt et al., 2019; Meilgaard, 1975
Acetic acid	175000	53.90 ± 24.18	76.43 ± 9.85	185.40 ± 15.11	0.00 ± 0.00	0.00 ± 0.00	0.00 ± 0.00	Vinegar	Meilgaard, 1975
Ethyl acetate	30000	1286.91 ± 100.37	224.00 ± 9.09	1490.07 ± 112.17	0.04 ± 0.00	0.01 ± 0.00	0.05 ± 0.00	Fruity, solvent, sweet	Meilgaard, 1975; Verstrepen et al., 2003
2-Methyl-1-butyl acetate	1600	56.23 ± 3.64	42.96 ± 3.03	131.01 ± 14.39	0.04 ± 0.00	0.03 ± 0.00	0.08 ± 0.01	Fruity, banana	Meilgaard, 1975
Isobutyl acetate	1600	35.34 ± 5.67	27.20 ± 2.36	89.86 ± 4.58	0.02 ± 0.00	0.02 ± 0.00	0.06 ± 0.00	Fruity, apple	Holt et al., 2019; Meilgaard, 1975
Isoamyl acetate	1600	1159.21 ± 86.62	826.98 ± 57.70	3285.19 ± 208.79	0.72 ± 0.05	0.52 ± 0.04	**2.05 ± 0.13**	Banana, pear, fruity	
2-Phenylethyl acetate	3800	4852.53 ± 484.81	5983.59 ± 298.69	20833.80 ± 1342.59	**1.28 ± 0.13**	**1.57 ± 0.08**	**5.48 ± 0.35**	Honey, floral, rose	Meilgaard, 1975; Verstrepen et al., 2003
Ethyl butanoate	400	190.00 ± 23.92	14.81 ± 0.69	215.67 ± 4.49	0.47 ± 0.06	0.04 ± 0.00	0.54 ± 0.01	Pineapple, fruity	Meilgaard, 1975
Ethyl hexanoate	210	1003.34 ± 136.80	280.59 ± 31.84	2733.83 ± 257.87	**4.78 ± 0.65**	**1.34 ± 0.15**	**13.02 ± 1.23**	Apple, aniseed, fruity	Holt et al., 2019; Meilgaard, 1975
Ethyl octanoate	900	6333.75 ± 639.42	5149.46 ± 411.24	14817.18 ± 1070.51	**7.04 ± 0.71**	**5.72 ± 0.46**	**16.46 ± 1.19**	Apple, sweet, pineapple	Meilgaard, 1975; Verstrepen et al., 2003
Ethyl decanoate	1500	235.11 ± 49.92	394.12 ± 69.70	5665.70 ± 2076.41	0.16 ± 0.03	0.26 ± 0.05	**3.78 ± 1.38**	Apple, grape, fatty, solvent	Meilgaard, 1975
Ethyl dodecanoate	1000	47.84 ± 9.48	117.44 ± 30.58	669.27 ± 249.49	0.05 ± 0.01	0.12 ± 0.03	0.67 ± 0.25	Waxy, fatty	
Ethyl undecanoate	1000	1.85 ± 2.54	2.32 ± 3.18	38.22 ± 22.59	0.00 ± 0.00	0.00 ± 0.00	0.04 ± 0.02	Fatty Acids, pear, grassy	
4-Vinylguaiacol	300	111.06 ± 14.00	1991.67 ± 164.40	1893.40 ± 116.16	0.37 ± 0.05	**6.64 ± 0.55**	**6.31 ± 0.39**	Clove, spicy, smoky	

The values show the mean of the replicates (*n* = 5) and their standard deviation (SD). OAV > 1 are indicated in bold.

Among higher alcohols, no one exceeded their odor thresholds (OAV < 1), suggesting that these compounds had little individual impact on aroma despite being present at relatively high concentrations. In contrast, several acetate and ethyl esters presented OAVs higher than 1. The hybrid DR1 showed higher OAVs for 2-phenylethyl acetate (5.48 ± 0.35), ethyl hexanoate (13.02 ± 1.23) and ethyl octanoate (16.46 ± 1.19) than the parental strains. Furthermore, the concentration of isoamyl acetate and ethyl decanoate in parental strains Lal 2 and AQ 3504 beers do not reach the threshold values, while the hybrid DR1 exhibited OAVs above 1, 2.05 ± 0.13 for isoamyl acetate and 3.78 ± 1.38 for ethyl decanoate. The parental strain AQ3504 showed the highest OAV for 4-vinylguaiacol (6.64 ± 0.55), followed closely by DR1 (6.31 ± 0.39), while Lal2 remained below the perception threshold (0.37 ± 0.05).

To explore the relationship between aroma-active compounds and sensory perception, a PCA integrating OAVs with CATA frequencies was performed (Figure 8).

**FIGURE 8:**
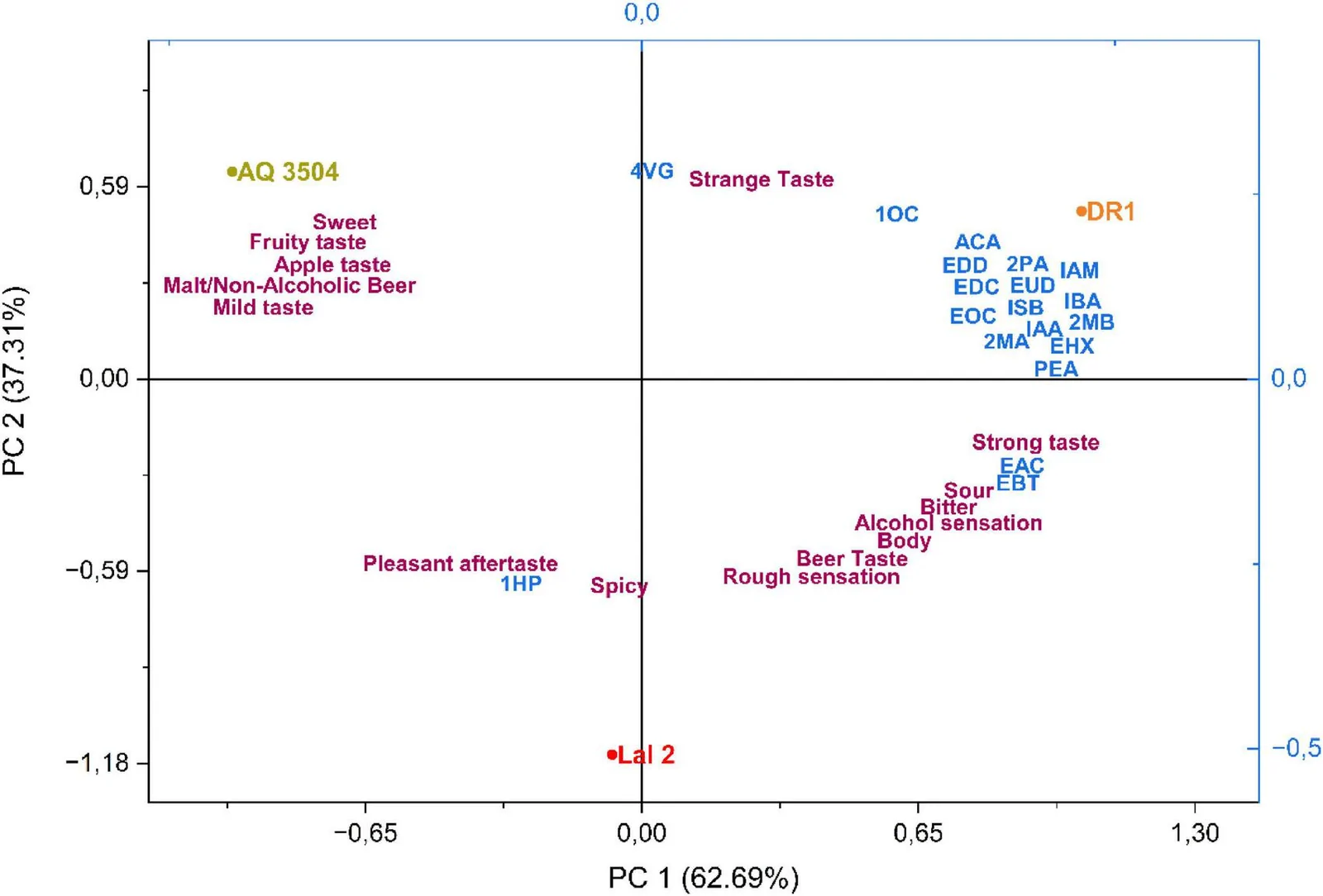
Principal Component Analysis (PCA) illustrating the relationship between odor activity values (OAVs) of volatile compounds and the sensory profile of the three beers studied. Beer samples (Lal 2, AQ 3504 and DR1) are represented by colored circles, volatile compounds by blue abbreviations, and sensory descriptors by purple labels. The first two principal components (PC1 and PC2) accounted for 62.69% and 37.31% of the total variance, respectively. Compound abbreviations: IBO, isobutanol; IAO, isoamyl alcohol; MBO, 2-methylbutanol; HPO, 1-heptanol; OCO, 1-octanol; PEO, 2-phenylethyl alcohol; ACD, acetic acid; ETA, ethyl acetate; MBA, 2-methyl-1-butyl acetate; IBA, isobutyl acetate; IAA, isoamyl acetate; PEA, 2-phenylethyl acetate; EBE, ethyl butanoate; EHE, ethyl hexanoate; EOE, ethyl octanoate; EDE, ethyl decanoate; ENE, ethyl dodecanoate; EUE, ethyl undecanoate; and 4VG, 4-vinylguaiacol.

PC1 mainly separated AQ 3504 from DR1, whereas PC2 differentiated Lal 2 from the other two beers. DR1 was close to the majority of aroma-active esters, particularly ethyl hexanoate, ethyl octanoate, 2-phenylethyl acetate, isoamyl acetate and ethyl decanoate, indicating that these compounds largely defined its aroma profile. AQ 3504 was strongly associated with 4-vinylguaiacol and sensory descriptors as sweet, fruity taste, apple taste, mild taste and malt/non-alcoholic beer. In contrast, Lal 2 was mainly associated with 1-heptanol and the descriptor pleasant aftertaste, while remaining separated from the ester-rich aroma profile observed for DR1.

## 4 Discussion

This study analyzed the kinetic, metabolic, volatile and sensory characteristics of the interspecific hybrid DR1 (*S. cerevisiae* × *S. paradoxus*) generated by rare-mating to reduce the alcohol content of the final beer and improve its aromatic profile. This work builds on our previous studies, in which we revealed the high phenotypic diversity among brewing-compatible yeast strains from different species and subpopulations highlighting non-conventional strains that exhibited lower ethanol production associated with a deficiency in maltose and maltotriose uptake (Roldán-López et al., 2025) and we identified strains of *S. cerevisiae, S. uvarum* and *S. paradoxus* with valuable volatile profiles for brewing applications (Roldán-López et al., 2026). Several approaches have been proposed to reduce ethanol content in beer, including physical dealcoholizing and biological approaches based on alternative yeasts, adaptive evolution, or hybridization (Blanco et al., 2016; Winans, 2022).

The objective of the present work was to develop a *Saccharomyces* hybrid capable of moderately reducing ethanol production while preserving the fermentation performance and sensory characteristics associated with conventional brewing yeasts. In the context of brewing, the ethanol reductions achieved by DR1 (21% in representative wort and 17.5% in industrial wort), corresponding to approximately 1.3–1.4% v/v less ethanol than the commercial parent, are considered moderate because they preserve fermentation performance and beer-like sensory characteristics while providing a meaningful decrease in alcohol content. In this context, the selection of parental strains for hybrid generation is a critical step, since the objective is to combine complementary technological traits in the resulting hybrid (Zavaleta et al., 2024). The kinetic and metabolic differences observed among the 28 strains initially evaluated confirmed that sugar utilization is a key determinant of ethanol production and supported the selection of parents with traits suited to the development of a reduced alcohol, aroma-enhanced brewing strain.

The hybrid performance therefore suggests that rare-mating may have combined transport and hydrolytic functions from the parental strains in a way that supports balanced sugar utilization. However, the phenotype also suggests that some maltotriose uptake capacity may have been lost or diluted during hybrid formation, since maltotriose utilization in brewing yeasts is often more fragile and less robust than maltose utilization and depends on a narrower set of transporters and regulatory determinants (Horák, 2013; Vidgren et al., 2005). The constitutive expression of the MALx1, AGT1, MPH2 and MPH3 transporters is responsible for the metabolism of maltose and maltotriose. However, it is demonstrated that the agt1 transporter (encoded by the MAL11 variant or AGT1 gene) is the transporter required for the uptake of the trisaccharide maltotriose, even in strains harboring multiple active MALx1, MPH2 or MPH3 loci (Alves et al., 2008). In this context, previous studies on maltose and maltotriose transport systems indicate that maltotriose utilization is typically the first trait to deteriorate when transporter repertoires are reduced or regulatory compatibility is incomplete, whereas maltose consumption is usually more stable because it is supported by a broader and more redundant genetic background (Horák, 2013; Magalhães et al., 2016). Thus, the phenotype observed in DR1 may reflect a hybrid in which maltose-related functions were retained more efficiently than maltotriose transport, although this interpretation should be confirmed by genomic and transcriptional analyzes of the inherited *MAL* loci.

Parental strains were strategically selected to combine complementary technological traits through rare-mating. Industrial *S. cerevisiae* strains with strong maltose/maltotriose-positive fermentation profiles (WS 34/70, Lal 1, Lal 2, BBG) were paired with low-fermentation non*-cerevisiae Saccharomyces* strains (AQ 1905, AQ 3196, AQ 3504) to achieve reduced ethanol production while maintaining brewing-relevant aroma characteristics. Specifically, Lal 2 (*S. cerevisiae*) was chosen for its proven fermentative capacity and industrial aroma profile, while AQ 3504 (*S. paradoxus*) contributed to reduced alcohol fermentation and novel aromatic potential at brewing temperatures (Nikulin et al., 2020). In our previous work, we demonstrated the potential of *S. paradoxus* for the production of low-alcohol beer, as it is not able to consume maltotriose and has a variable ability to consume maltose, depending on the strain (Roldán-López et al., 2025). Similarly, in a subsequent study, it was shown that the *S. paradoxus* strains yHDPN429 and CBS432 exhibited a VOM profile similar to that found in commercial hybrids, but with a 1.5% (v/v) reduction in ethanol content due to the consumption of maltose but not maltotriose (Roldán-López et al., 2026).

The resulting DR1 hybrid (Lal 2 × AQ 3504) successfully integrated these traits, achieving the primary objective of ethanol reduction (∼1.5% v/v lower than the *S. cerevisiae* Lal 2) through intermediate sugar metabolism. Despite achieving lower ethanol yield than the commercial parental Lal 2 strain (∼1.5% v/v reduction), DR1 exhibited increased glycerol production relative to both parental strains, yielding nearly 1.2 g/L more glycerol than Lal 2. This elevation is particularly advantageous for reduced alcohol beers, where glycerol enhances body, mouthfeel, and overall sensory balance (Zhao et al., 2015). Such hybrid-specific traits align with broader biotechnological goals, including climate-adaptive wine production where elevated glycerol mitigates high-alcohol effects (Tilloy et al., 2014).

This glycerol increase, alongside the hybrid’s intermediate sugar metabolism, underscores rare-mating’s potential to generate strains with balanced fermentation profiles for reduced-alcohol beverages. The DR1 phenotype demonstrates how interspecific hybridisation can redirect carbon flux toward desirable non-ethanol metabolites while preserving essential brewing functionality, offering a non-GMO route to innovate reduced alcohol beer production with improved sensory attributes. A complete carbon balance could not be established because biomass and several potential fermentation products were not quantified. Carbon not recovered as ethanol or glycerol may have been allocated to CO_2_, biomass, organic acids, or other metabolites, but dedicated carbon-flux analyzes would be required to determine the contribution of each pathway.

DR1 exhibited heterosis for aroma production, with substantially increased concentrations of several aroma-active esters, including isoamyl acetate, 2-phenylethyl acetate, ethyl hexanoate and ethyl octanoate, compared with both parental strains. However, 4-vinylguaiacol (4-VG) (typically considered undesirable in brewing) was produced by DR1, mirroring the *S. paradoxus* parent AQ 3504 and reflecting the POF+ phenotype common in interspecific hybrids due to inherited functional *PAD1* or *FDC1* genes (Gallone et al., 2016; Nikulin et al., 2018). While the *S. cerevisiae* parent Lal 2 exhibits minimal 4-VG (POF-), traditional selection has favored clean-flavored POF- strains. Notably, the POF+ phenotype in such hybrids can be reversed through sporulation, offering a route to mitigate this trait while retaining desirable aroma enhancements. Despite these sensory limitations, DR1 retained several beer-like attributes also associated with Lal 2, supporting its potential as a prototype for further optimization rather than as a ready-to-use industrial strain.

A sensory analysis supported DR1’s technological interest while highlighting areas for refinement. The hybrid beer retained essential traditional beer attributes (characteristic bitterness and conventional flavor profile) closely matching that produced by the commercial *S. cerevisiae* Lal 2 strain. However, panelists detected an “atypical” flavor that reduced overall acceptability, likely attributable to elevated concentrations of certain aroma compounds perceived as negative at high levels, or specifically to 4-vinylguaiacol off-flavor production characteristic of the POF+ phenotype. By contrast, beer from the *S. paradoxus* parent AQ 3504 was rated as overly sweet due to incomplete fermentation and high residual sugars. Ultimately, DR1 bridges these extremes, producing a reduced alcohol beer sensorially proximate to commercial standards but enriched with novel aromatic complexity that merits further optimisation for broad consumer acceptance. The lower purchase intention observed for DR1 beers appears to be mainly related to their sensory profile. Penalty analysis showed that atypical and strong flavor attributes negatively affected consumer acceptance, whereas conventional beer flavor, pleasant aftertaste, body and alcohol sensation positively influenced liking. The reduced perception of typical beer flavor in DR1 beers is likely associated with their distinct VOM profile compared with conventional commercial beers. Although no direct correlation analysis was performed between individual VOMs and sensory scores, the PCA integrating volatile and sensory datasets showed that samples characterized by higher levels of ethanol-derived esters clustered with positive beer-like sensory attributes, supporting the relationship between aroma composition and consumer perception.

Furthermore, the sensory relevance of each VOM is relative to its perception threshold. Due to the importance of the matrix, this study has only considered those compounds for which the threshold in beer is well known. Previous studies have demonstrated generally high threshold values in beer (Meilgaard, 1975). The OAV analysis clearly demonstrated that not all VOMs quantified contributed equally to beer aroma.

Higher alcohols represented some of the most abundant compounds detected, but their direct contribution to aroma was limited. They exhibited OAVs well below unity in all beers due to their relatively high odor thresholds. This agrees with previous studies reporting that higher alcohols generally require concentrations above approximately 300,000 μg/L before generating undesirable solvent-like or alcoholic notes (Zhang et al., 2019), whereas at lower concentrations they mainly act as precursors for acetate ester synthesis rather than as direct aroma-active compounds (Pires et al., 2014).

In contrast, acetate and ethyl esters represented the main contributors to aroma perception. In this study, one acetate ester and one ethyl ester were found to exceed their respective sensory perception thresholds exclusively in the hybrid and not in the parental strains. Although higher alcohols provide precursors for ester formation, the increased production of ethanol-derived esters observed in DR1 was not accompanied by proportional changes in higher alcohol concentrations. This suggests that factors other than precursor availability, such as altered acetyltransferase activity or ester metabolism, may contribute to the volatile profile of this hybrid. Among aroma-active compounds, the largest differences were associated with isoamyl acetate, 2-phenylethyl acetate, ethyl hexanoate and ethyl octanoate, all of which displayed higher OAV values in DR1 and are known contributors to fruity and floral beer aromas. Overall, these results indicate that the sensory profile of DR1 was not explained by a general increase in volatile production but rather by the selective enhancement of a small group of highly aroma-active esters. This observation is consistent with reports showing that interspecific *Saccharomyces* hybrids can display transgressive ester production, generating concentrations that exceed those of both parental strains and leading to novel aromatic phenotypes (Bellon et al., 2015; Zavaleta et al., 2024). Such improvements in aroma-active ester production highlight the potential of non-GMO hybridisation as a strategy for developing novel brewing yeasts with differentiated sensory profiles.

The industrial suitability of the DR1 hybrid depends on the target beer style. Its incomplete maltotriose utilization can be advantageous for the production of reduced-alcohol beers, resulting in a 21% reduction in ethanol production while retaining residual sugars (Bellut and Arendt, 2019). In addition, the POF+ phenotype could be advantageous for specific beer styles, such as German wheat beers and several Belgian-style ales, in which clove-like phenolic notes are considered characteristic and desirable (Gibson et al., 2017; Lehnhardt et al., 2026). Similarly, higher ester production is desirable in many Belgian-style ales, where fruity aroma is a defining characteristic (Gibson et al., 2017; Verstrepen et al., 2003). The enhanced banana and fruity flavor due to the isoamyl acetate perception is a key attribute in Hefeweizen beers (Şutea et al., 2025). The industrial potential of a yeast strain should always be evaluated according to the intended beer style.

Nevertheless, DR1 should be regarded as a promising prototype rather than a final industrial strain, because additional genomic and sensory optimisation will be required before industrial deployment. Only one main hybrid was characterized in depth, and complete genomic analysis will be required to clarify its subgenome composition, stability and the genetic basis of its maltotriose-negative and POF+ phenotypes. While the present study focused on phenotypic characterization, future genomic, transcriptomic and enzymatic analyses will be necessary to identify the molecular determinants underlying the hybrid’s sugar utilization phenotype and altered volatile metabolite production. In addition, although DR1 consistently reduced ethanol production relative to Lal 2, it should be considered a reduced-ethanol strain rather than a true low-alcohol beer yeast, and its moderate consumer acceptance indicates the need for further sensory optimization. Future work should focus on selecting POF− segregants or stabilized variants, adaptive laboratory evolution and backcrossing strategies to retain the desirable ethanol reduction and ester production while improving consumer acceptance.

In conclusion, this study demonstrates that rare-mating can be used to generate non-GMO interspecific brewing hybrids with modified sugar utilization patterns, reduced ethanol production, and distinctive aroma profiles. The results support the potential of hybridisation as a strain development strategy to address the increasing demand for beers with lower alcohol content and differentiated sensory properties. However, DR1 should be considered a proof-of-concept strain rather than a final industrial product. Future work should focus on genomic characterization, evaluation of long-term genetic stability, optimisation of flavor attributes and consumer acceptance, and the development of improved variants adapted to specific brewing applications. More broadly, the DR1 hybrid provides a useful model for investigating how interspecific hybridisation shapes fermentation performance and aroma production in industrial *Saccharomyces* yeasts.

## Data Availability

The datasets presented in this study can be found in the online repository Zenodo (https://doi.org/10.5281/zenodo.20082889).

## Footnotes

1
http://www.amdis.net/What_is_AMDIS/AMDIS_Literature_and_Downloads/amdis_literature_and_downloads
