# Supplementary material for: A non-GMO Saccharomyces cerevisiae × Saccharomyces paradoxus brewing hybrid moderately reduces ethanol production and modifies beer aroma
Source: Front Microbiol. 2026 Sep 29;17:1948430. doi: 10.3389/fmicb.2026.1948430 (PMC13659296; doi:10.3389/fmicb.2026.1948430)
Supplement: Supplementary file 1 [file Table_1.docx]

**Table S1**

GC-MS Conditions.

| GC-PARAMETERS | |
| --- | --- |
| Rate-temperature 1 | 5 °C/min-73 °C |
| waiting time 1 | 4 min |
| Rate-temperature 2 | 5 °C/min-192 °C |
| waiting time 2 | 0 min |
| Rate-temperature 3 | 20 °C/min-250 °C |
| waiting time 3 | 5 min |
| injector | splitless 250°C |
| transfer line | 270 °C |
| MS-PARAMETERS | |
| Transfer line temperature | 270 ◦C |
| Ion Source temperature | 230 ◦C |
| mass range (m/z) | 40-350 |
| Frequency(scan/sec) | 2.4 |
| GERSTEL PARAMETERS | |
| Incubation temperature | 45 °C |
| Incubation time | 20 min |
| Agitation time | 60 s |
| Agitator pause time | 1 s |
| Agitator speed | 350 rpm |
| Sample tray type | R32 LiqCooled/20-CVM |
| Vial penetration | 38 mm |
| Extraction time | 40 min |
| Injection penetration | 54.00 mm |
| Desorption time | 180 s |
| Pre-conditioning time | 30 min |
| Post-conditioning time | 0 min |
| Conditioning penetration | 43 mm |

**Table S2**

Analytes and ion models.

| Analytes | Rt | Ion models |
| --- | --- | --- |
| Isobutanol  (2-methyl propanol) | 1.67970385 | 74 |
| Isoamyl alcohol  (3-methyl butanol) | 2.87250769 | 55 |
| 2-Methylbutanol | 2.91653846 | 56 |
| 2-Ethylhexanol | 12.03252 | 83 |
| 1-Heptanol | 9.9183 | 70 |
| 1-Octanol | 14.0357615 | 84 |
| 1-Dodecanol | 28.2684042 | 111 |
| 1-Tetradecanol | 33.0622 | 97 |
| 2-Phenylethanol | 16.2536769 | 122 |
| 2.4-Di-tert-butylphenol | 29.2724885 | 191 |
| 3-Methylbutanal | 1.8717125 | 71 |
| Decanal | 20.2322591 | 112 |
| Dodecanal | 26.5719889 | 140 |
| 4-Methylbenzaldehyde | 14.236 | 119 |
| Acetic acid | 1.55425 | 60 |
| 2-Methylpentanoic acid | 29.7595292 | 117 |
| Tetradecanoic acid | 34.9306889 | 185 |
| Ethyl acetate | 1.60292692 | 70 |
| 2-Methyl-1-butyl acetate | 6.80925769 | 74 |
| Isobutyl acetate | 3.60463043 | 73 |
| Isoamyl acetate | 6.74174615 | 87 |
| 2-Phenylethyl acetate | 21.9973 | 104 |
| Ethyl butanoate | 4.31688077 | 88 |
| Ethyl hexanoate | 10.9285308 | 99 |
| Butyl hexanoate | 18.030119 | 117 |
| Ethyl octanoate | 20.0791808 | 127 |
| Propyl octanoate | 23.1657286 | 145 |
| Isoamyl octanoate | 27.540868 | 171 |
| Ethyl nonanoate | 23.2993038 | 141 |
| 2-Methylbutyl octanoate | 27.6216682 | 184 |
| Isobutyl octanoate | 24.8245111 | 127 |
| Butyl decanoate | 30.0238105 | 173 |
| Ethyl 9-decenoate | 26.0667308 | 152 |
| Butyl 9-decenoate | 29.8416722 | 135 |
| Ethyl 9-tetradecenoate | 35.29762 | 209 |
| Ethyl 9-hexadecenoate | 39.02775 | 194 |
| 2-Phenylethyl 3-methylbutanoate | 36.6419409 | 104 |
| Ethyl decanoate | 26.3077231 | 155 |
| Propyl decanoate | 28.6816316 | 173 |
| 2-Methylbutyl decanoate | 32.4074842 | 224 |
| Ethyl dodecanoate | 31.23375 | 183 |
| Ethyl undecanoate | 28.7918056 | 101 |
| Ethyl tetradecanoate | 35.58115 | 213 |
| Ethyl pentadecanoate | 37.600605 | 101 |
| Ethyl hexadecanoate | 39.3350577 | 284 |
| Ethyl octadecanoate | 41.1700115 | 312 |
| Ethyl 9-octadecenoate | 40.9839385 | 222 |
| Ethyl 7-octenoate | 19.58189 | 124 |
| Ethyl 3-phenylpropionate | 24.8009444 | 133 |
| Ethyl linoleate | 40.9386133 | 308 |
| 1-(1-ethoxyethoxy)pentane | 10.0263273 | 145 |
| Chloromethyl 8-chlorododecanoate | 31.0690286 | 138 |
| Diethyl azelate | 33.3163308 | 199 |
| Ethyl 3-ethoxybenzoate | 29.5206846 | 194 |
| 4.5-Dimethyl-1-hexene | 32.1681591 | 197 |
| 1.3-Di-tert-butylbenzene | 21.8185154 | 175 |
| Dihydrobenzofuran | 21.046175 | 120 |
| 6-Hydroxy-4'-methoxyflavone | 18.4748538 | 267 |
| Damascenone | 25.813564 | 190 |
| Pyrrolidinobutiothiophenone | 21.0174958 | 153 |
| 4-Vinylguaiacol | 23.7779115 | 150 |
| 4-Fluorobenzaldehyde | 9.29867308 | 124 |

**Table S3**

AMDIS Analytical settings.

| Settings | Values |
| --- | --- |
| Minimum match factor | 85% |
| Retention time window | 0.20 min |
| maximum penalty | 20 |
| No. of RT in Library | 10 |
| Threshold | medium |
| Adjacent peak subtraction | 1 |
| Resolution | Low |
| Sensitivity | medium |
| Shape requirements | Low |

**Table S4**

Confirmed auxotrophic yeast strains collection generated by selecting auxotrophic strains using selective α-AA (Lys-). 5-FOA (Ura-) and 5-FAA (Trp-) media.

|  | Lys^-^ | Ura^-^ | Trp^-^ |
| --- | --- | --- | --- |
|  | Α-AA medium | 5-FOA medium | 5-FAA medium |
| WS 34/70 | 0 | 0 | 0 |
| Lal 1 | 0 | 0 | 0 |
| Lal 2 | 1 | 0 | 0 |
| BBG | 0 | 0 | 0 |
| CGD | 0 | 0 | 0 |
| AQ 3196 | 7 | 4 | 2 |
| AQ 1905 | 0 | 0 | 0 |
| AQ 3936 | 0 | 0 | 0 |
| AQ 3504 | 6 | 0 | 2 |

**Table S5**

Endpoint metabolic analysis of the DR1 hybrid (*S. cerevisiae x S. paradoxus*) and the parental strains Lal 2 *(S. cerevisiae*) and AQ 3504 (*S. paradoxus*) in brewing conditions with representative brewing wort. Maltotriose (%), maltose (%) and glucose (%) consumption and ethanol (% v/v) and glycerol (g/L) production is shown.

| Yeast strain | Maltotriose consumption (%) | Maltose consumption (%) | Glucose consumption (%) | Ethanol production (% v/v) | Glycerol production (g/L) |
| --- | --- | --- | --- | --- | --- |
| Lal 2  (S.c) | 91.22 ± 1.38 | 99.32 ± 0.33 | 99.03 ± 0.05 | 6.57 ± 0.05 | 2.26 ± 0.36 |
| AQ 3504  (S.p) | 1.60 ± 1.41 | 2.51 ± 1.26 | 100 | 1.12 ± 0.01 | 0.64 ± 0.09 |
| DR1  (S.c x S.p) | 0 | 96.26 ± 0.23 | 99.00 ± 0.31 | 5.20 ± 0.10 | 2.69 ± 0.69 |

**Table S6**

Endpoint metabolic analysis of the DR1 hybrid (*S. cerevisiae* x *S. paradoxus*) and the parental strains Lal 2 (*S. cerevisiae*) and AQ 3504 (*S. paradoxus*) in industrial brewing conditions with industrial brewing wort. Maltotriose (%), maltose (%) and glucose (%) consumption and ethanol (% v/v) and glycerol (g/L) production is shown.

| Yeast strain | Maltotriose consumption (%) | Maltose consumption (%) | Glucose consumption (%) | Ethanol production (% v/v) | Glycerol production (g/L) |
| --- | --- | --- | --- | --- | --- |
| Lal 2  (S.c) | 86.77 ± 1.10 | 98.21 ± 0.45 | 100 | 7.53 ± 0.10 | 2.33 ± 0.26 |
| AQ 3504  (S.p) | 2.53 ± 2.87 | 1.61 ± 2.85 | 100 | 1.31 ± 0.04 | 1.50 ± 0.05 |
| DR1  (S.c x S.p) | 7.47 ± 0.83 | 96.85 ± 0.06 | 100 | 6.21 ± 0.10 | 3.46 ± 0.40 |
